# Polymer-based chemical-nose systems for optical-pattern recognition of gut microbiota†

**DOI:** 10.1039/d2sc00510g

**Published:** 2022-04-26

**Authors:** Shunsuke Tomita, Hiroyuki Kusada, Naoshi Kojima, Sayaka Ishihara, Koyomi Miyazaki, Hideyuki Tamaki, Ryoji Kurita

**Affiliations:** Health and Medical Research Institute, National Institute of Advanced Industrial Science and Technology 1-1-1 Higashi Tsukuba Ibaraki 305-8566 Japan s.tomita@aist.go.jp r.kurita@aist.go.jp; DBT-AIST International Laboratory for Advanced Biomedicine (DAILAB), DBT-AIST International Center for Translational & Environmental Research (DAICENTER) Japan; Bioproduction Research Institute, National Institute of Advanced Industrial Science and Technology Japan; Cellular and Molecular Biotechnology Research Institute, National Institute of Advanced Industrial Science and Technology Japan; JST ERATO Nomura Microbial Community Control Project, University of Tsukuba Japan; Faculty of Pure and Applied Sciences, University of Tsukuba Japan

## Abstract

Gut-microbiota analysis has been recognized as crucial in health management and disease treatment. Metagenomics, a current standard examination method for the gut microbiome, is effective but requires both expertise and significant amounts of general resources. Here, we show highly accessible sensing systems based on the so-called chemical-nose strategy to transduce the characteristics of microbiota into fluorescence patterns. The fluorescence patterns, generated by twelve block copolymers with aggregation-induced emission (AIE) units, were analyzed using pattern-recognition algorithms, which identified 16 intestinal bacterial strains in a way that correlates with their genome-based taxonomic classification. Importantly, the chemical noses classified artificial models of obesity-associated gut microbiota, and further succeeded in detecting sleep disorder in mice through comparative analysis of normal and abnormal mouse gut microbiota. Our techniques thus allow analyzing complex bacterial samples far more quickly, simply, and inexpensively than common metagenome-based methods, which offers a powerful and complementary tool for the practical analysis of the gut microbiome.

## Abstract 2

A biomimetic ‘chemical-nose’ composed of twelve block copolymers with aggregation-induced emission units is presented, which can detect sleep disorder in mice from a small amount of microbiome samples (<10 μg) in a short time (<30 min).

## Graphical abstract

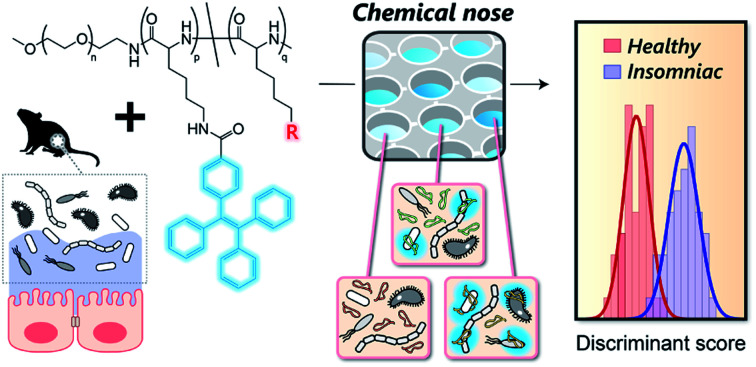

## Introduction

A wide variety of microorganisms [∼100 trillion (10^14^) cells of more than 1000 species] inhabits the human gastrointestinal tract.^1^ Recent studies have demonstrated that these microbial populations, known as the ‘gut microbiome’, interact with the human host and are thus closely related to health and disease development.^2^ Microbial imbalances, or dysbiosis, have been observed in patients with various diseases, including chronic sleep disruption, obesity, allergies, autism-spectrum disorders, and cancer.^3–5^ Therefore, understanding and controlling gut microbiota is critical for health management and disease treatment.

The current standard method to analyze the gut microbiome is metagenomic analysis, which involves sequencing the 16S ribosomal RNA (rRNA) gene amplicons or the entire genome of each bacteria in the microbiota. Although metagenomics is a powerful tool that enables investigating the diversity and structure of the entire microbiotic community of the gut ecosystem and the genomic and metabolic capability of its constituents,^6,7^ it requires expensive next-generation sequencers and a high level of expertise for data acquisition and analysis, in addition to significant amounts of general resources (labor and time). Many medical and biological applications require a more rapid assessment of gut microbiome samples using limited equipment. Additionally, the interpretation and insights obtained *via* metagenomics may not correspond to the actual state of the microbiota due to biases resulting from sample pretreatment, sequencing, and data analysis.^6,7^

Exploring diagnostic and therapeutic approaches based on differences between the gut microbiota of healthy and diseased individuals detected *via* metagenomic analysis has revealed that, despite the high complexity of the gut ecosystem, gut microbiota exhibit characteristic compositional patterns at various taxonomic rank levels, such as genus-level enterotypes^8,9^ or the phylum-level *Firmicutes*/*Bacteroidetes* (*F*/*B*) ratio,^10–12^ which correlate with the status of the host (*e.g.*, disease status and physical conditions). Inspired by these studies, we envisioned that such discrete compositional states could be recognized *via* a sequencing-independent ‘chemical nose’ sensing strategy.

The chemical-nose strategy is an analytical concept that mimics the sensory mechanisms of animals by combining arrays of molecular probes and pattern recognition algorithms. Using a library of molecular probes that exhibit varying affinities for samples of interest, chemical noses generate characteristic patterns of information that reflect the entire sample *via* comprehensive interactions between the molecular probes and the sample components.^13,14^ The chemical-nose method has been shown to be applicable to the detection of purified pathogenic bacteria in the context of diagnosing infectious diseases.^15–24^ As with olfaction, the essence of this strategy is that samples can be analyzed comparatively from the generated response patterns even when the sample composition is unknown. Thus, the chemical-nose strategy is suitable for determining whether a sample is normal or for classifying its state. Indeed, we^25–27^ and other groups^16,28–32^ have already demonstrated that this strategy can identify the states of complex biological samples, such as serum,^26^ cell lysates,^28,32^ and cell secretions,^25,27^ as well as volatile organic compounds produced by bacteria,^16,29–31^ but not gut microbiome samples.

**Fig. 1:**
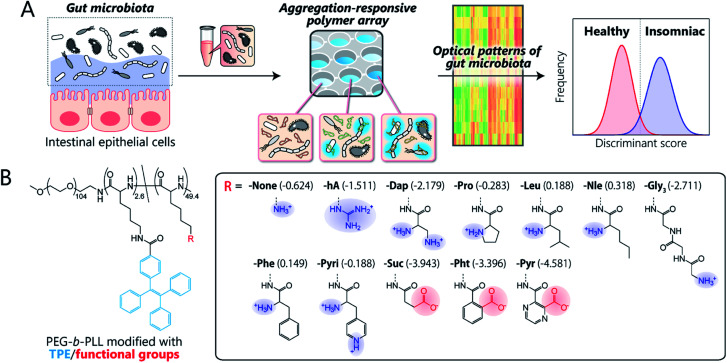
Workflow and synthetic library for the optical-pattern recognition of gut microbiota. (A) The collected samples of mouse gut microbiota are analyzed using a chemical nose composed of aggregation-responsive polymers to generate fluorescence-response patterns that reflect the characteristics of the entire gut microbiota, which are then statistically analyzed using pattern-recognition algorithms. (B) The molecular structures of PEG-*b*-PLL block copolymers modified with TPE fluorophores and various functional groups. The log *P* values of the head groups are shown in parentheses.

Here, we present the first chemical-nose platform for recognizing gut microbiota. To analyze these largely unknown and obscure samples, we have created a synthetic library of charged block copolymers functionalized with aggregation-induced emission (AIE) fluorophores. Our chemical-nose systems, which comprise up to twelve structurally diverse and environmentally sensitive polyethylene glycol-*block*-poly-l-lysine (PEG-*b*-PLL) derivatives, generate characteristic ‘fluorescence response patterns’ through various interactions with the surfaces of intestinal bacteria (Fig. 1A). The generated response patterns are analyzed using pattern-recognition algorithms, which allows identifying gut-derived bacterial strains at different taxonomic levels (strains/species/phylum) and of mixed intestinal bacterial suspensions used as artificial models of the gut microbiota of obese and healthy individuals. Furthermore, this system is able to accurately distinguish the gut microbiota of healthy mice from those of insomniac mice.

## Results

### Design and characterization of a polymer library

Key to designing the polymer library for the differentiation of highly complex gut microbiota, which consist of over 1000 bacterial species,^1^ was the incorporation of two properties: (i) high chemical diversity to allow the recognition of the surface characteristics, size, and morphology of the intestinal bacteria *via* a variety of interactions, and (ii) sharp turn-on emission characteristics to allow reproducible transduction of the microenvironments of the bacterial surfaces into optical signals.

Fig. 1B illustrates the library of block copolymers synthesized to meet the criteria. For the sensitive and selective extraction of bacterial characteristics, PEG-*b*-PLL was chosen as the scaffold material because its high density of functionalizable sites can be expected to facilitate multi-contact interaction with the bacteria.^14,33^ The amino groups of PEG-*b*-PLL were partially modified with the AIE luminogen tetraphenylethene (TPE). The TPE-functionalized PEG-*b*-PLL (-None) was expected to emit fluorescence upon binding to bacterial surfaces due to the restricted intramolecular rotation of the central olefin stator of the TPE molecule.^34^ To provide high structural diversity, the remaining amino groups of -None were (i) guanidinized to enhance hydrogen-bonding ability (-hA), (ii) modified with amino acids with different aliphatic and aromatic groups or additional amino groups (-Dap, -Pro, -Nle, -Leu, -Phe, and -Pyri) or a hydrophilic tripeptide (-Gly_3_), or (iii) modified with acid anhydrides for cationic-to-anionic charge inversion (-Suc, -Pht, and -Pyr). As these modifications cover a wide chemical space, we hypothesized that this polymer array could potentially be able to recognize the complex features of bacterial surfaces, which consist of lipopolysaccharides, lipids, peptidoglycans, and proteins,^35^ and transduce this surface information into highly sensitive AIE signals due to the low background fluorescence of the TPE fluorophore (for details regarding the synthesis and characterization of the polymers, see Sections 3 and 4 in the ESI†).

**Fig. 2:**
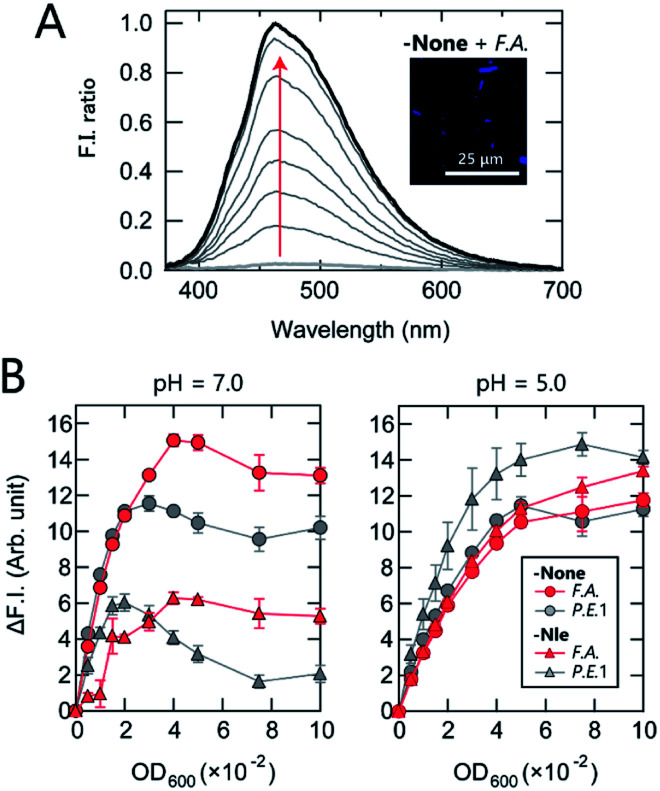
Characterization of representative polymers. (A) Fluorescence spectra of -None (150 nM) upon addition of *F.A.* (OD_600_ = 0–0.05) in 20 mM MOPS buffer (pH = 7.0); *λ*_ex_ = 330 nm. Inset: fluorescence image of *F.A.* treated with -None. (B) Binding isotherms for -None and -Nle (150 nM) upon addition of *F.A.* and *P.E.*1 in 20 mM MOPS (pH = 7.0) or 20 mM acetate buffer (pH = 5.0) with 150 mM NaCl; *λ*_ex_/*λ*_em_ = 330 nm/460 nm. The values shown represent mean values ±1 SE from three independent experiments.

First, the AIE response of TPE-functionalized PEG-*b*-PLL (-None) in the presence of bacteria was examined. While -None was nearly non-emissive in aqueous solution [20 mM 3-morpholinopropanesulfonic acid (MOPS) buffer; pH = 7.0], the addition of the anaerobic, intestinal bacterium *Anaerostipes caccae* (phylum *Firmicutes*; *F.A.*; Table 1) enhanced the fluorescence emission nearly 40-fold (Fig. 2A). This response suggests that -None was bound to or aggregated on the bacteria primarily through electrostatic interactions with the dense array of negatively charged teichoic acids present on the surface of the Gram-positive *F.A.*,^35^ causing restriction of the intramolecular motion of TPE. The AIE of -None was also examined by fluorescence microscopy images, in which blue emission from TPE was observed only on the surface of *F.A.* [Fig. 2A inset and S1A†; *Escherichia coli* DH5a (phylum *Proteobacteria*; *P.E.*1) is also shown in Fig. S1B†]. These results are consistent with previous microscopic observations of TPE derivatives for pattern-recognition-based bacteria sensing.^18,22^ The sharp turn-on fluorescence of our TPE-functionalized polymer was expected to provide higher experimental reproducibility than our previously reported dansyl-fluorophore-modified polymers^36^ due to the high signal-to-background ratio of the former, which is a significant advantage in constructing high-precision chemical noses.

**Table 1:** Gut-derived bacterial strains used for initial testing

*Phylum*	Genus species	Abbr
*Firmicutes*	*Anaerostipes caccae*	*F.A.*
	*Blautia hydrogenotrophica*	*F.B.*
	*Clostridium citroniae*	*F.C.*
	*Eubacterium fissicatena*	*F.E.*
	*Ruminococcus gauvreauii*	*F.R.*
	*Lactococcus lactis*	*F.L.*1
	*Lactobacillus helveticus*	*F.L.*2
*Bacteroidetes*	*Bacteroides dorei*	*B.B.*1
	*Bacteroides oleiciplenus*	*B.B.*2
	*Bacteroides clarus*	*B.B.*3
	*Bacteroides coprophilus*	*B.B.*4
*Actinobacteria*	*Bifidobacterium faecale*	*A.B.*1
	*Bifidobacterium thermophilum*	*A.B.*2
	*Bifidobacterium longum*	*A.B.*3
*Proteobacteria*	*Escherichia coli* (DH5α)^a^	*P.E.*1
	*Escherichia coli* (JM109)^a^	*P.E.*2

a Strain name.

We then investigated the relationship between the polymer structure and its fluorescence response at physiological ionic strength (Fig. 2B). At pH = 7.0, -None exhibited a similar increase in fluorescence intensity in the presence of both *F.A.* and *P.E.*1 at an optical density at 600 nm (OD_600_) <0.03, but its intensity was higher in *F.A.* than in *P.E.*1 at higher OD_600_ values. The fluorescence response of hydrophobic -Nle to *F.A.* reached almost a plateau at OD_600_ >0.04, while it decreased significantly at OD_600_ >0.02 in the case of *P.E.*1. In addition, the responses to bacteria varied markedly depending on the pH value; the responses of the polymers in 20 mM acetate buffer (pH = 5.0) were similar, except in the case of -Nle/*P.E.*1. The pH dependence of the fluorescence responses was attributed primarily to the protonation of the carboxyl groups on the bacterial surface and the amino groups of the polymers when the pH value decreased from 7.0 to 5.0, especially in the case of -Nle (Section 4 in the ESI†). These complex responses are most likely due to differences in the chemical structure of the polymers; this fact was expected to favor the use of our polymer system for extracting features of bacterial samples.

### Differentiation of gut-derived bacterial strains

Before seeking to evaluate real microbiome samples, we initially tested whether our polymers could generate unique optical-response patterns for its constituent, gut-derived bacterial culture strains (Table 1). The 16 bacterial strains chosen covered the predominant phyla in the gut microbiota, *Actinobacteria*, *Bacteroidetes*, *Firmicutes*, and *Proteobacteria*, which constitute more than 97% of the human gut microbiome.^37^ For the sensing procedure, each bacterial suspension was added to an array consisting of the polymers (150 nM) in 20 mM MOPS buffer (pH = 7.0) or 20 mM acetate buffer (pH = 5.0) with 150 mM NaCl. The fluorescence responses from each bacterial strain/polymer combination were recorded using two different channels (Ch1: *λ*_ex_/*λ*_em_ = 330 nm/480 nm; Ch2: *λ*_ex_/*λ*_em_ = 360 nm/530 nm), which provided a dataset of 48-dimensional fluorescence patterns (12 polymers × 2 pH values × 2 channels). A preliminary study found that applying background subtraction (*I* − *I*_0_), which is usually required to improve the accuracy of chemical noses,^38^ was not necessary (Fig. S2 and Dataset 1†), presumably due to the low background fluorescence of TPE. This feature is advantageous for the construction of a practical system, as it saves labor and time.

**Fig. 3:**
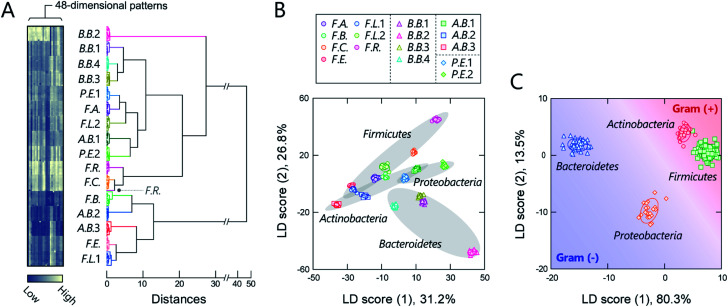
Optical pattern recognition of gut-derived bacteria. (A) Heat map and the resulting HCA dendrogram of the fluorescence-response patterns of the 16 different intestinal bacterial strains (OD_600_ = 0.04). For each analyte, 11 independent experimental values are shown. Each pattern in the heat map corresponds to the end of the dendrogram. (B, C) LDA score plot for the intestinal bacterial strains (OD_600_ = 0.04), wherein the analytes are labelled according to (B) species and (C) phylum. The ellipsoids represent the confidence intervals (±1 SD) for each analyte.

The responses are summarized visually in the form of a heat map in Fig. 3A and S3 (for the raw data, see Dataset 2†). Applying the intestinal bacterial suspensions to our chemical nose obviously produced a variety of fluorescence response patterns. An unsupervised hierarchical clustering analysis (HCA), in which the calculated distance between elements corresponds to the similarities in the response patterns,^13^ exhibited well-separated clusters (except for one case of *F.R.*), indicating that the differences among the patterns of the 16 bacterial strains evaluated are statistically significant (Fig. 3A).

The discriminative information was then statistically evaluated using linear discriminant analysis (LDA), a supervised pattern-recognition algorithm that provides graphical output describing the similarity and the classification ability of the data.^13^ The LDA score plot (Fig. 3B and S4†), in which each point represents the fluorescence-response pattern of a single analyte in the chemical nose, again showed that the clusters were well-separated. The discrimination accuracy was quantitatively evaluated using two different tests [a leave-one-out cross-validation test (the so-called jackknife test) and a holdout test]; 100% accuracy in the identification of the bacterial strains was successfully achieved in both tests (Dataset 2†). Furthermore, a minimal system with sufficient accuracy could be constructed using two selected polymers (Section 5 in the ESI†).

Intriguingly, clusters were observed at the phylum level, which is a taxonomic rank much higher than the species level (Fig. 3B), even though the bacterial phylotypes have not been well proven to correlate with their surface properties. This trend was also observed in an unsupervised principal component analysis (PCA; Fig. S5†). To evaluate the potential of this phylum-level feature extraction in more detail, a meta-analysis was performed with phylum labeling of the data for each bacterium. As expected, clusters corresponding to the four phyla were well segregated in the linear discriminant score plot (Fig. 3C), and high accuracy was again achieved in both the jackknife test (99%) and the holdout test (100%) (Dataset 2†). Recent studies have revealed that gut microbiota exhibit phylum- or genus-level compositional patterns,^8–12^ suggesting that our chemical nose, which can recognize phylum-level features of intestinal bacterial strains, is suitable for providing the response patterns of gut microbiota. Our chemical nose also succeeded in recognizing differences between bacteria that are classified into the same ‘species’ but differ in the lower ‘strain’ classification (*i.e.*, eight *Escherichia coli* strains; Section 6 in the ESI†).

Based on the HCA results, the subsequent studies used a lower number of combinations while maintaining a sufficiently high performance of the sensor elements, *i.e.*, six polymers (-None, -Dap, -Gly_3_, -Leu, -Phe, and -Pht), two pH values, and Ch1 (for details, see Section 5 in the ESI†).

### Classification of models of gut microbiota associated with obesity

**Fig. 4:**
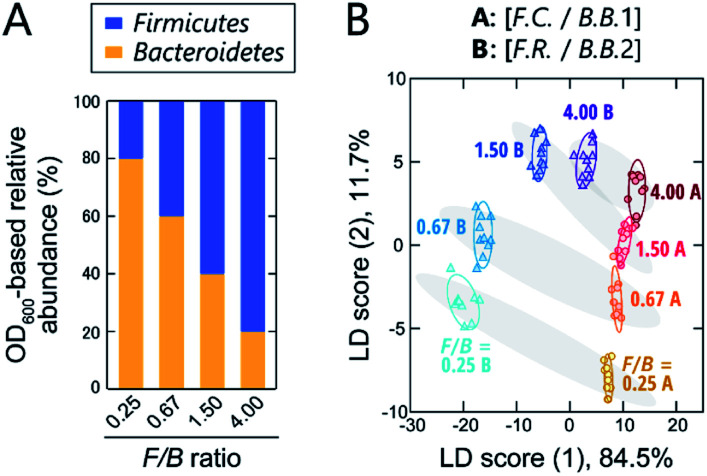
Optical pattern recognition of model bacterial mixtures corresponding to the microbiota of obese and healthy individuals. (A) Relative abundance of intestinal bacterial strains in samples with different *Firmicutes*/*Bacteroidetes* (*F*/*B*) ratios. (B) LDA score plot for the intestinal bacterial mixtures. For each analyte, 10 independent experimental values are shown. Ellipsoids represent the confidence intervals (±1 SD) for each analyte.

Although still controversial, the *F*/*B* ratio of gut microbiota is well known to vary between healthy humans and those with lifestyle diseases, such as obesity and type II diabetes. Thus, the gut microbiota composition, even at the phylum level, represents a potential biomarker or new therapeutic target for such diseases.^10–12^ To investigate the applicability of our chemical nose to *F*/*B*-ratio classification, tests were performed using model samples that simulated the gut microbiota of obese and healthy individuals. Based on a recent report,^39^ we prepared gut-microbiota models consisting of two intestinal bacterial strains that differ in their *Firmicutes* and *Bacteroidetes* content ratios (Fig. 4A). These model samples were intended to cover the range from underweight people (*F*/*B* ≈ 0.70) to obese people (*F*/*B* ≈ 1.54).^39^

The prepared model samples were applied to a chemical-nose system. Statistical analysis of the fluorescence response patterns (Fig. S6 and Dataset 3†) using LDA revealed that the eight clusters were distributed without overlap in the two-dimensional space (Fig. 4B) with high discriminant accuracies; 98% for the jackknife test and 94% for the holdout test (Dataset 3†).

Interestingly, the clusters of the *F.C.*/*B.B.*1 mixtures and the *F.R.*/*B.B.*2 mixtures, although composed of different bacterial strains, exhibited similar migration with changing *F*/*B* ratio. Consistent with this result, when LDA was performed at *F*/*B* ratios where individual strains were not distinguished, high identification accuracy was maintained (94% and 100%, respectively; Dataset 3†). This predictive behavior of clusters corresponding to the systematic changes in microbial mixtures could possibly be used in the future to quantify *F*/*B* ratios of real samples, where species-level composition alterations are more complex, using regression analysis based on machine-learning techniques, such as support vector machine.^13^ The ability of this chemical nose to accurately recognize the subtle differences between the model bacterial mixtures demonstrates its promise for applications to real samples (*vide infra*). Our chemical nose also has the potential to detect trace amounts of gut-derived bacteria spiked into a real microbiome, suggesting different forms of use for this system (Section 7 in the ESI†).

### Application to the diagnosis of insomnia in mice

One potential application of gut-microbiome measurements is the diagnosis or routine monitoring of health status as well as that of serious disease development.^40,41^ We thus carried out a proof-of-concept study to demonstrate the ability of this chemical-nose system to detect a deterioration of health condition in mice *via* optical pattern recognition of the microbiota in mouse feces.

**Fig. 5:**
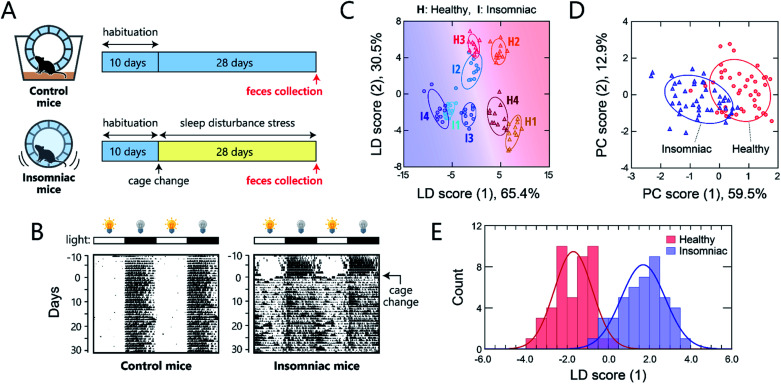
Optical pattern recognition of mouse gut microbiota. (A) Mouse feces collection procedure. After 10 days of habituation in a normal cage, the mice in the insomnia group were transferred to the sleep-disturbance cage on day 0; feces were collected from the insomnia and control group after 28 days. (B) Representative double-plot actograms for the control mice and those subjected to sleep disturbance. The black regions represent periods during which the mouse was rotating the wheel. The light/dark cycles are shown as white and black bars, respectively, above the actograms. (C) LDA score plot for the feces from individual mice (20 μg mL^−1^) and (D) PCA score plot of the fluorescence response patterns for the feces from the healthy and insomniac mice (20 μg mL^−1^). The ellipsoids represent the confidence intervals (±1 SD) for each analyte. For each analyte, 11 independent experimental values are shown. (E) Histogram of the LDA scores for mouse gut microbiota; the red and blue curves are normal distributions fitted to the full data.

For this study, we used a sleep-disorder model to prepare mice with an adverse health condition;^42^ we have recently reported that such sleep disruption causes memory dysfunction and anxiety-like behavior.^43^ A continuous stress, namely, disturbed sleep, was imposed on 8 week-old mice by confining them inside a rotating wheel^42^ for 28 days after a 10 day-habituation period in a normal cage with a rotating wheel (Fig. 5A and S7†). In the normal cages, the nocturnal mice were significantly more active at night, whereas the stressed mice ran on the wheel almost all day immediately after exposure to the stress, causing sleep fragmentation (Fig. 5B). Homogeneous microbiota suspensions were prepared as follows: Fecal samples, collected on day 28 from the healthy or insomniac mice (*n* = 4 for both), were suspended in phosphate buffer saline (PBS), centrifuged to remove the soluble fraction, and filtered through a 40 μm mesh to remove large aggregates. The thus obtained gut microbiome samples (4 individuals × 2 conditions) were diluted to 200 μg mL^−1^ with distilled water and subsequently used in experiments. In contrast to bacterial culture suspensions, we attempted to simplify the measurement procedure and hence improve the experimental practicality by choosing the mass concentration instead of the optical density.

As with the axenic cultures of gut-derived bacteria (Fig. 2), the addition of the mouse gut microbiome samples to the TPE-functionalized PEG-*b*-PLL markedly enhanced its fluorescence emission (Fig. S8†). After confirming this response, we used the chemical nose to generate the fluorescence response patterns (Fig. S9†).

The samples of the individual mice could be discriminated with little overlap in the two-dimensional score plot (Fig. 5C) and 92% accuracy in the jackknife test, even though these individuals were raised in the same environment prior to treatment. Using this labeling, the clusters of the healthy and insomniac mice were also separated, suggesting that the differences between the responses induced by the presence or absence of stress were higher than those among individuals. HCA analysis showed a slight mixture of the two-state analytes (Fig. S10†), whereas PCA suggested a potentially distinguishable difference between their distributions as expected (Fig. 5D). Indeed, LDA analyses showed a good separation between the healthy and insomniac groups, with only a marginal overlap in the histogram of the discriminant scores (Fig. 5E). The two-tailed, unpaired Student's *t*-test indicated a *p* value of <1.8 × 10^−3^ (Fig. S11†). Consistent with this differentiation, both the jackknife test and the holdout test indicated relatively high reliability (91% for both tests; Dataset 4†). We also successfully constructed a minimal sensor for mouse gut microbiota with an accuracy of up to 97% (Section 5 in the ESI†). Although the gut microbiota most likely contains bacteria in various growth stages, our results suggest that from a practical perspective, the impact on the analysis in our chemical noses can be considered negligible.

## Discussion

In the present study, we constructed a synthetic library of block copolymers functionalized with AIE fluorophores to establish a chemical-nose strategy for gut-microbiota sensing. Our chemical nose composed of TPE-functionalized PEG-*b*-PLLs is capable of (i) identifying the species and phyla of 16 intestinal bacterial strains and the strains of eight *Escherichia coli*, (ii) classifying artificial models of gut microbiota associated with obesity, and (iii) detecting sleep disorder through comparative evaluation of mouse gut microbiota. This study represents the first successful demonstration of the application of a chemical-nose strategy to real, complex gut microbiota (mouse fecal samples), which consist of more than 1000 bacterial species.^1^

The achievements presented should be attributed largely to the development of a suitable novel polymer library for a microbiome-targeted chemical nose. The twelve TPE-functionalized PEG-*b*-PLLs cover a wide chemical space and bind the target analytes in a multi-contact manner that combines electrostatic, hydrophobic, π–π, and hydrogen-bonding interactions. Since bacteria contain charged polysaccharides and hydrophobic lipids, as well as a variety of polymers and proteins, on their external cell surfaces,^35^ individual PLL-based polymers interact with the entire components in a complex manner. TPE, which emits light in response to changes in its molecular rotational motion,^34^ converts the inhibition of its motion due to polymer binding into a fluorescence signal, which contains information not only on the amount of polymer binding but also on the motion-inhibition efficiency of the binding site. In addition, TPE provides a high signal-to-background ratio, which in turn leads to high reproducibility and greatly simplifies the sensing procedure. These features most likely enabled the recognition of the patterns of characteristic microbiota compositions correlated with the condition of the host.^8–12^

The chemical-nose strategy has previously been applied to detect purified pathogenic bacteria using libraries of fluorescent probe molecules,^16,18,20–22^ polymers,^23^ nanomaterials,^24^ and supramolecular complexes.^15,19^ However, chemical noses focusing on gut-derived bacteria have not yet been reported. Our system can discriminate gut-derived bacteria at not only the species, but also at the phylum level, which represents a much higher phylogenetic rank (Fig. 3C). The outer surfaces of Gram-positive bacteria [Gram (+); *Actinobacteria* and *Firmicutes*] are composed of peptidoglycans, which are typically intercalated with covalently attached anionic teichoic acids, while those of Gram-negative bacteria [Gram (−); *Bacteroidetes* and *Proteobacteria*] are surrounded by a negatively charged outer membrane containing lipopolysaccharides.^35^ These different outer structures produce large physicochemical differences, which our chemical nose would be expected to recognize, as outlined in previous reports.^19,20,24^ However, the differences among the outer structures of bacteria with the same gram-stainability are not well understood. Therefore, the ability to extract information regarding such unknown differences is a distinguishing feature of our system.

We further demonstrated that our method is applicable to real mouse gut microbiota. Although the major phyla that dominate the gut microbiota could be distinguished (Fig. 3C), in practice it is not clear which bacterial species in the gut microbiota were predominantly recognized by the chemical nose. Regardless, our results clearly indicate that this new method can accurately discriminate the characteristics of real gut microbiome samples associated with different health conditions in the host mice without using metagenomic analysis. To further verify and validate the effectiveness of our method for simple diagnosis, particularly in the routine monitoring of specific individuals, it is essential in the future to conduct comparative analyses of our method with metagenomics and serum metabolomics, which are correlated to the development of insomnia,^44^ and also to investigate if the chemical nose is generally applicable to mice of different ages, those reared in different environments, and even humans.

The versatility and limitations of the chemical-nose strategy are not yet fully understood. The lack of information regarding individual bacteria in the output can be considered an advantageous feature of this method as well as a weakness compared to metagenomic analysis. Nevertheless, our current chemical nose is capable of measuring relatively small samples (<10 μg) in a short time (<30 min) using only common analytical instrumentation, and comparative analysis can be performed based on the overall characteristics of the samples even when the details of the target microbiota are unknown. The human microbiome, which includes all the microorganisms inhabiting the human body has recently attracted increasing interest among researchers ranging from basic scientists to medical professionals.^45^ Additionally, microorganisms are ubiquitous in the environment and play a crucial role in controlling not only biogeochemical cycles,^46^ but also agricultural productivity and sustainability.^47–49^ Therefore, we expect our approach to complement metagenomic analysis to offer a powerful platform for the simple, accurate, and high-throughput characterization of microbiota in a wide range of fields, from medicine and healthcare to ecology and agriculture.

## Data availability

The datasets supporting this article have been uploaded as part of the ESI.†

## Author contributions

Conceptualization: S. T., H. K., K. M., H. T. and R. K.; formal analysis: S. T.; funding acquisition: S. T., H. K., H. T. and R. K.; data curation, investigation: S. T., H. K., N. K., S. I., K. M. and H. T.; resources: S. T., H. K., N. K., K. M. and R. K.; visualization: S. T., N. K. and K. M.; supervision: H. T. and R. K.; writing – original draft, methodology: S. T. and H. K.; writing – review & editing: all authors.

## Conflicts of interest

There are no conflicts to declare.

## Supplementary Material

SC-013-D2SC00510G-s001

SC-013-D2SC00510G-s002
