# Supplementary material for: Polymer-based chemical-nose systems for optical-pattern recognition of gut microbiota
Source: Chem Sci. 2022 Apr 26;13(20):5830–7. doi: 10.1039/d2sc00510g (PMC9132137; doi:10.1039/d2sc00510g)
Supplement: SC-013-D2SC00510G-s002 [file SC-013-D2SC00510G-s002.pdf]

Electronic Supplementary Material (ESI) for Chemical Science.
This journal is © The Royal Society of Chemistry 2022

Supplementary Information

Polymer-based chemical-nose systems for optical-pattern recognition of gut microbiota

Shunsuke Tomita,*[‡ab] Hiroyuki Kusada,[‡c] Naoshi Kojima,[b] Sayaka Ishihara,[b] Koyomi Miyazaki,[d] Hideyuki Tamaki,[ce] and Ryoji Kurita*[abf]

[a] Health and Medical Research Institute; [b] DBT-AIST International Laboratory for Advanced Biomedicine (DAILAB); [c] Bioproduction Research Institute; [d] Cellular and Molecular Biotechnology Research Institute, National Institute of Advanced Industrial Science and Technology, 1-1-1 Higashi, Tsukuba, Ibaraki 305-8566, Japan. [e] JST ERATO Nomura Microbial Community Control Project; [f] Faculty of Pure and Applied Sciences, University of Tsukuba, 1-1-1 Tennodai, Tsukuba, Ibaraki 305-8573, Japan; [‡]These authors contributed equally.

*Shunsuke Tomita, Ryoji Kurita

Email: s.tomita@aist.go.jp (S.T.), r.kurita@aist.go.jp (R.K.).

Section 1: Materials and Methods

Preparation of bacterial samples

Gut-derived bacteria. All anaerobic microbes used in this study (Table S1) were purchased from culture collections (DSMZ and JCM) and cultivated as described elsewhere.[1] In brief, the cultivation was performed using Gifu Anaerobic Medium (GAM, Nissui Pharmaceutical Co. Ltd) with N_2/CO_2 (80:20, v/v) as the headspace gas at 37 °C under anaerobic conditions. Eight *Escherichia coli* strains (Table S2) were grown in Luria–Bertani medium at 37 °C. Cell densities were measured as the optical density at 600 nm (OD_{600}) using a Nanodrop 2000c spectrophotometer (Thermo Fisher Scientific). The cells were stored anaerobically as glycerol stocks (final glycerol concentration 20%) in 10 mL glass vials at -80 °C until used.

Mouse gut microbiome. The mouse models of sleep disorder were prepared according to a slightly modified literature procedure.[2,3] Male C3H-HeN mice were purchased from Japan SLC Inc., housed under a 12 h light : 12 h dark cycle (lights on at 08:00) at a controlled ambient temperature of 24 ± 1 °C, and provided with food (CE-2, CLEA Japan Inc.) and water *ad libitum* throughout the study. Starting at 8 weeks of age (day -10), the mice were maintained individually in plastic cages containing paper animal bedding and running wheels (SW-15, Melquest) for 10 days before being divided into the healthy control group and induced-sleep-disorder group. Mice in the normal cage group (healthy mice) were housed individually in SW-15 plastic cages with paper bedding and running wheels from day -10 to 28. The remaining mice (insomniac mice) were transferred to sleep-disturbance cages (SW-15-SD, Melquest) where the mouse was constantly exposed to the stress of unstable ground as it is trapped in an unanchored running wheel. On day 28, the individual mice were transferred to plastic cages, and their feces were collected in microtubes immediately after excretion. The collected samples were rapidly frozen in liquid nitrogen and stored at -80 °C. Wheel-running activity was monitored and recorded in 1 min bins using a Chronobiology Kit (Stanford Software Systems) and displayed as double-plot actograms, as described previously.[4] The experiments were conducted in accordance with the guidelines for the Care and Use of Laboratory Animals at the National Institute of Advanced Industrial Science and Technology (AIST), and the Animal Care and Use Committee at AIST approved the study protocol (approval no. 2011–056b).

Characterization of the polymers

Acid–base titration of polymers. A pH meter (SevenCompact, Mettler-Toledo) equipped with pH probes (InLab Expert Pro-ISM, Mettler-Toledo) was calibrated using three standard buffers (pH = 4.0, 6.9, and 9.2) prior to use. The solution temperature was controlled with a water bath, and the solution pH was recorded every 15 s. Solutions containing the synthesized tetraphenylethene (TPE)-functionalized polyethylene glycol-*block*-poly-L-lysine (PEG-*b*-PLLs) (15 µM; concentrations of the ionic groups were 0.7–0.8 mM), 10 mM NaCl, and 2.0 mM NaOH (**-Pyr**) or 2.0 mM HCl (all other polymers) were titrated with a solution containing 10 mM NaCl and 30 mM HCl (**-Pyr**) or 30 mM NaOH (all other polymers) at 25 °C while stirring under a nitrogen atmosphere. The apparent acid dissociation constant (pKa) at 25 °C was determined based on the Henderson–Hasselbalch equation.

Fluorescence of the polymers at various pH values. Fluorescence measurements were performed using a Cytation5 Imaging Reader (BioTek Instruments, Inc.). Solutions (120 µL) containing 150 nM of the TPE-functionalized PEG-*b*-PLLs in mixed

buffers of 2-morpholinoethanesulfonic acid (MES) and 3-morpholinopropanesulfonic acid (MOPS) (20 mM for both, pH = 4.5 to 9.0) were prepared in each well of a 96-half-well NBS™ black microplate (Corning Inc.) using a PIPETMAX liquid handling system (Gilson Inc.). After incubation (35 °C, 10 min), the fluorescence spectrum ($\lambda_{ex}/\lambda_{em}$ = 330 nm/372–700 nm) or the fluorescence intensity ($\lambda_{ex}/\lambda_{em}$ = 330 nm/460 nm) was recorded at 35 °C.

Fluorescence responses of the polymers to bacteria. Prior to the study, the stock solution of gut-derived bacteria was thawed at 4 °C and centrifuged at 6000 g (10 °C, 10 min). The supernatant was removed, and distilled water was added to reach OD_{600} = 0.5 for the bacteria. Fluorescence measurements were performed using a Cytation5 Imaging Reader. Solutions (120 µL) containing 150 nM TPE-functionalized PEG-*b*-PLLs and gut-derived bacteria (OD_{600} = 0–0.1) in 20 mM MOPS buffer (pH = 7.0) or 20 mM acetate buffer (pH = 5.0) with/without 150 mM NaCl were prepared in each well of a 96-half-well NBS™ black microplate using a PIPETMAX liquid handling system. After incubation (35 °C, 10 min), the fluorescence spectrum ($\lambda_{ex}/\lambda_{em}$ = 330 nm/372–700 nm) or the fluorescence intensity ($\lambda_{ex}/\lambda_{em}$ = 330 nm/460 nm) were recorded at 35 °C. For fluorescence microscopy imaging, solutions containing 15 µM **-None** and gut-derived bacteria (OD_{600} = 1.0) in 20 mM MOPS (pH = 7.0) with 150 mM NaCl were placed on a glass slide, and then imaged using a fluorescence cell imager (ZOE™; Bio-Rad Laboratories Inc.) at $\lambda_{ex}/\lambda_{em}$ = 390 nm/446 nm.

Chemical-nose sensing

Gut-derived bacteria. The stock solution of gut-derived bacteria was thawed at 4 °C and centrifuged at 6000 g (10 °C, 10 min). The supernatant was removed and distilled water was added to reach OD_{600} = 0.5 for the bacteria. *Obesity model bacteria mixtures*. Two of the four different gut-derived bacteria were mixed in distilled water as indicated in Table S3. *Mouse gut microbiota*. Homogeneous microbiome suspensions were prepared by combining the methods provided in two reports;[5,6] phosphate buffer saline (PBS) was added to fecal samples collected on day 28 from healthy or insomniac mice (n = 4 for both) to give a concentration of 40 mg/mL. The solution was mixed by vortexing for 1 min and allowed to stand at 4 °C for 5 min; this process was repeated several times. To remove the soluble fraction, the resulting suspension was centrifuged at 8000 g (4 °C, 10 min), the supernatant was removed, and PBS was added. This process was repeated twice. A homogeneous suspension of the gut microbiome was then obtained by filtration with a sterile sieving device (pluriStrainer®, mesh size 40 µm, pluriSelect) to remove large aggregates, followed by a further 200-fold dilution with distilled water.

For the analysis of intestinal bacterial strains, the *Escherichia coli* strains and mouse gut microbiota, aliquots (108 µL) of solutions containing TPE-functionalized PEG-*b*-PLLs (167 nM) and 167 mM NaCl in 22.2 mM MOPS buffer (pH = 7.0) or 22.2 mM acetate buffer (pH = 5.0) were deposited in the wells of a 96-half-well plate using a PIPETMAX system. After incubation (35 °C, 10 min), the fluorescence intensity was recorded using two different channels (Ch1: $\lambda_{ex}/\lambda_{em}$ = 330 nm/480 nm; Ch2: $\lambda_{ex}/\lambda_{em}$ = 360 nm/530 nm). Subsequently, aliquots (12 µL) of the samples were added to each well, and the fluorescence intensity was recorded after incubation (35 °C, 10 min). For other analyses, aliquots (17.5 µL) of solutions containing TPE-functionalized PEG-*b*-PLLs (214 nM) and 214 mM NaCl in 28.5 mM MOPS buffer (pH = 7.0) or 28.5 mM acetate buffer (pH = 5.0) were deposited in the wells of a 384-well NBS™ black microplate (Corning Inc.) using a PIPETMAX system. After incubation (35 °C, 10 min), the fluorescence intensity was recorded using two different channels (Ch1: $\lambda_{ex}/\lambda_{em}$ = 330 nm/480 nm; Ch2: $\lambda_{ex}/\lambda_{em}$ = 360 nm/530 nm). Subsequently, aliquots (7.5 µL) of the samples were added to each well, and the fluorescence intensity was recorded after incubation (35 °C, 10 min). These processes were performed at least six times for

distinct samples to generate a training data matrix. This training data matrix was processed using linear discriminant analysis (LDA), hierarchical clustering analysis (HCA) and principal component analysis (PCA) in SYSTAT 13 (Systat Inc.). For holdout testing, four fluorescence patterns out of ten or eleven for each analyte were separated from the training data matrix and used as a test data matrix. The test data were classified in groups generated by the remaining training matrix according to their shortest Mahalanobis distances. HCA dendrograms were created based on the Euclidean distances using the Ward method and a dataset standardized prior to analysis using the following equation: $z = (x - \mu)/\sigma$, where z is the standardized score, x the raw score, μ the mean of the population, and σ the standard deviation of the population.

Section 2: Figures and Tables

Table S1. Gut-derived bacterial strains used in this study.

Phylum	*Genus*	*Species*	*Strain*	Abbr.	Ref.
Firmicutes	*Anaerostipes*	*caccae*	JCM13470^T	*F.A.*	7
Firmicutes	*Blautia*	*hydrogenotrophica*	DSM10507^T	*F.B.*	8
Firmicutes	*Clostridium*	*citroniae*	DSM19261^T	*F.C.*	9
Firmicutes	*Eubacterium*	*fissicatena*	DSM3598T	*F.E.*	10
Firmicutes	*Ruminococcus*	*gauvreauii*	JCM14987^T	*F.R.*	11
Firmicutes	*Lactococcus*	*lactis*	JCM5805^T	*F.L.*1	12
Firmicutes	*Lactobacillus*	*helveticus*	JCM1004	*F.L.*2	13
Bacteroidetes	*Bacteroides*	*dorei*	JCM13471^T	*B.B.*1	14
Bacteroidetes	*Bacteroides*	*oleiciplenus*	JCM16102^T	*B.B.*2	15
Bacteroidetes	*Bacteroides*	*clarus*	JCM16067^T	*B.B.*3	16
Bacteroidetes	*Bacteroides*	*coprophilus*	JCM13818^T	*B.B.*4	16
Actinobacteria	*Bifidobacterium*	*faecale*	JCM7044	*A.B.*1	17
Actinobacteria	*Bifidobacterium*	*thermophilum*	JCM7033	*A.B.*2	18
Actinobacteria	*Bifidobacterium*	*longum*	JCM1222^T	*A.B.*3	18
Proteobacteria	*Escherichia*	*coli*	*DH5α*	*P.E.*1	-
Proteobacteria	*Escherichia*	*coli*	*JM109*	*P.E.*2	-

^{T} Type strain.

Table S2. *Escherichia coli* strains used in this study.

Phylum	*Genus*	*Species*	*Strain*	Source
Proteobacteria	*Escherichia*	*coli*	DH5α (same as *P.E.*1)	GMbiolab
Proteobacteria	*Escherichia*	*coli*	JM109 (same as *P.E.*2)	TaKaRa
Proteobacteria	*Escherichia*	*coli*	BL21 (DE3) pLysS	BioDynamics
Proteobacteria	*Escherichia*	*coli*	Rosetta 2 (DE3)	Novagen
Proteobacteria	*Escherichia*	*coli*	Origami2 (DE3)	Novagen
Proteobacteria	*Escherichia*	*coli*	top10	Invitrogen
Proteobacteria	*Escherichia*	*coli*	Rosetta-gami B (DE3)	Novagen
Proteobacteria	*Escherichia*	*coli*	EPI300	Epicentre

Table S3. Compositions of the model mixtures of bacteria associated with obesity used in this study.

	OD_{600} values for each gut-derived bacterium in the mixture			
F/B ratio:	0.25	0.67	1.50	4.00
F.C. or *F.R.*	0.08	0.16	0.24	0.32
*B.B.*1 *or B.B.*2	0.32	0.24	0.16	0.08

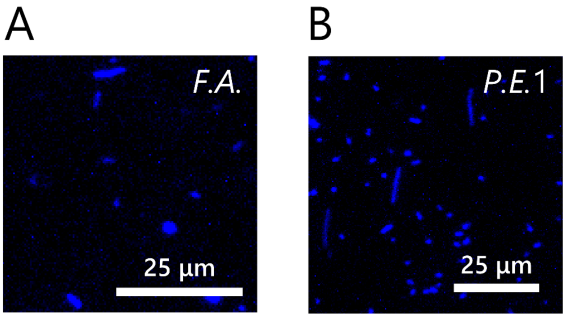

Fig. S1. Fluorescence microscopy images of *F.A.* (A) and *P.E.*1 (B) (OD_{600} = 1.0) in the presence of **-None** (15 µM) in 20 mM MOPS (pH = 7.0) with 150 mM NaCl. In both cases, blue fluorescence from TPE was only observed on the bacterial surfaces.

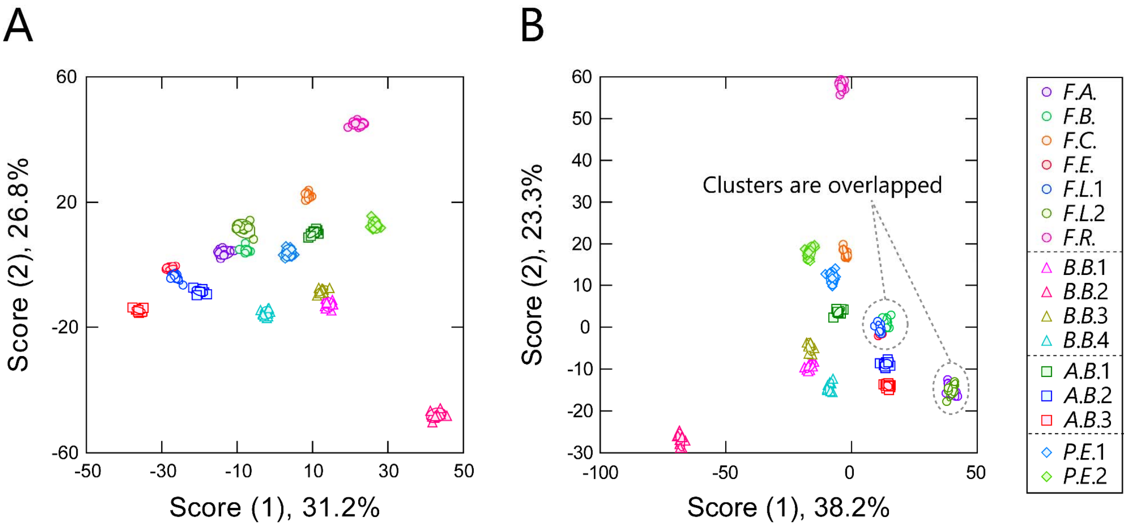

Fig. S2. Two-dimensional LDA score plots for gut-derived bacteria (OD_{600} = 0.04) obtained from the array consisting of 12 TPE-functionalized PEG-*b*-PLLs. The ellipsoids represent the confidence intervals (±1 SD) for each analyte. Two datasets, one consisting of the fluorescence response patterns of (A) the raw data (I) and the other of (B) the data after background subtraction (I–I_0), were subjected to LDA. For the raw data, the clusters of the 16 bacteria were spatially well separated, whereas using the I–I_0 data, several clusters overlapped (e.g., *F.A.* and *F.L.*2). The results of the jackknife test also indicated better accuracy for the raw data (100% for the raw data and 99% for I–I_0). These results suggest that background subtraction has a negative effect on the present system. Since many of TPE-functionalized PEG-*b*-PLLs exhibited low background fluorescence, the negative effect of the data variability in measuring I_0 may be greater than the positive effect of background cancellation.

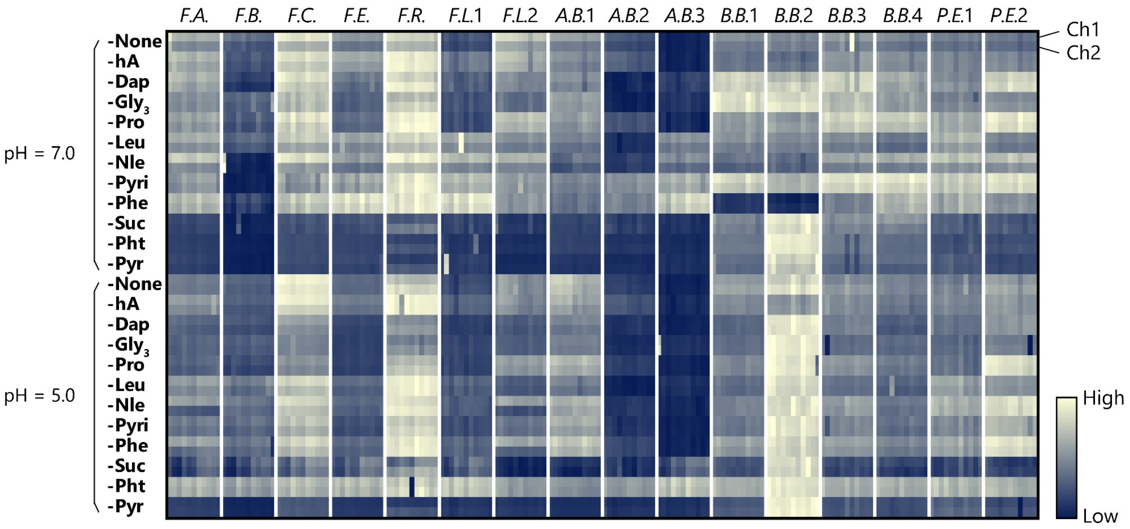

Fig. S3. Heat map of the fluorescence-response patterns of the 16 different intestinal bacterial strains (OD_{600} = 0.04). For each analyte, 11 independent experimental values are shown.

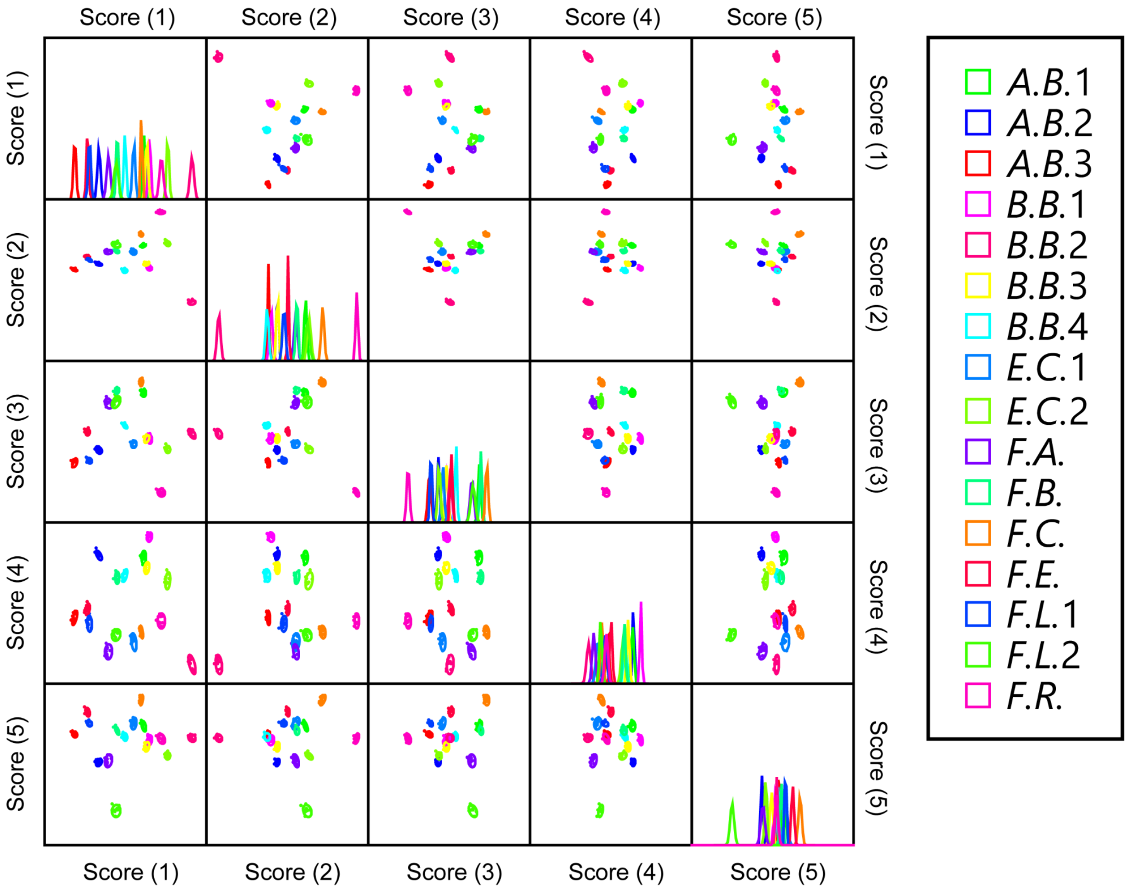

Fig. S4. LDA score plots of the first through fifth discriminant scores for the gut-derived bacteria (OD_{600} = 0.04) obtained using the array consisting of 12 TPE-functionalized PEG-*b*-PLLs. The data shown are the same as in Fig. 3. Many clusters were also isolated in the plots of score (3) through score (5), which accounted for only 14.1%, 7.6%, and 7.2% of the variation, respectively, indicating that our chemical nose succeeds in extracting various independent aspects of the gut-derived bacteria.

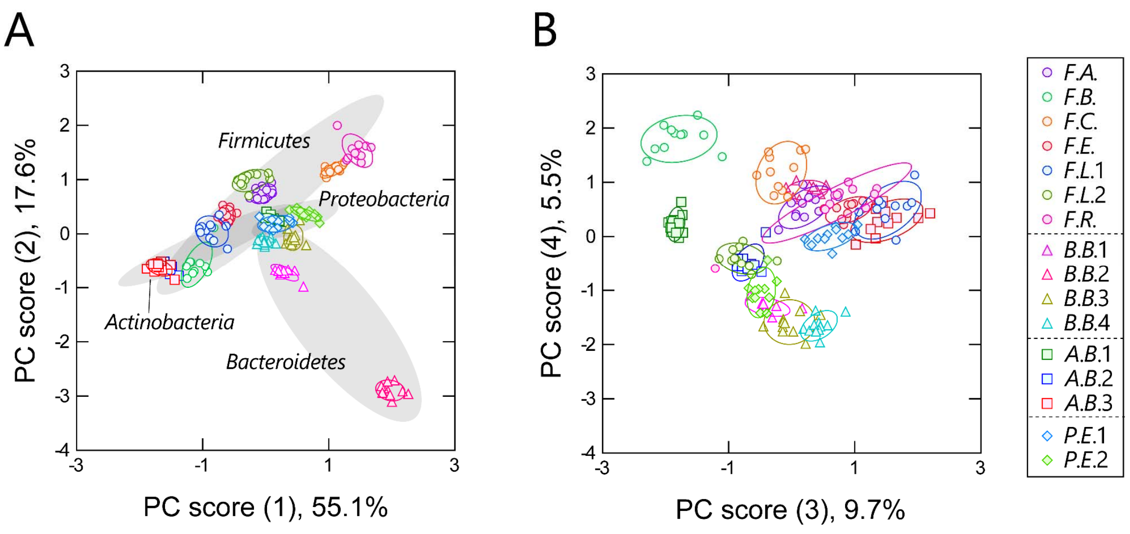

Fig. S5. PCA score plots for the intestinal bacterial strains (OD_{600} = 0.04) using the array consisting of 12 TPE-functionalized PEG-*b*-PLLs. (A) Score (1) vs. score (2); (B) score (3) vs. score (4). In the plot of (A), although the clusters overlapped slightly in comparison with the LDA score plot (Fig. 3B), their distribution was similar and there was a tendency for clusters to form even at the phylum level. In addition, the overlapping clusters in the plot of (A) were separated in the plot of (B), e.g., *A.B.*2/*A.B.3* and *P.E.*1/*A.B.1*, indicating high cross-reactivity for our array. The absence of phylum level clusters in the HCA dendrogram (Fig. 3A) suggests that factors other than gram-stainability are more dominant in pattern generation.

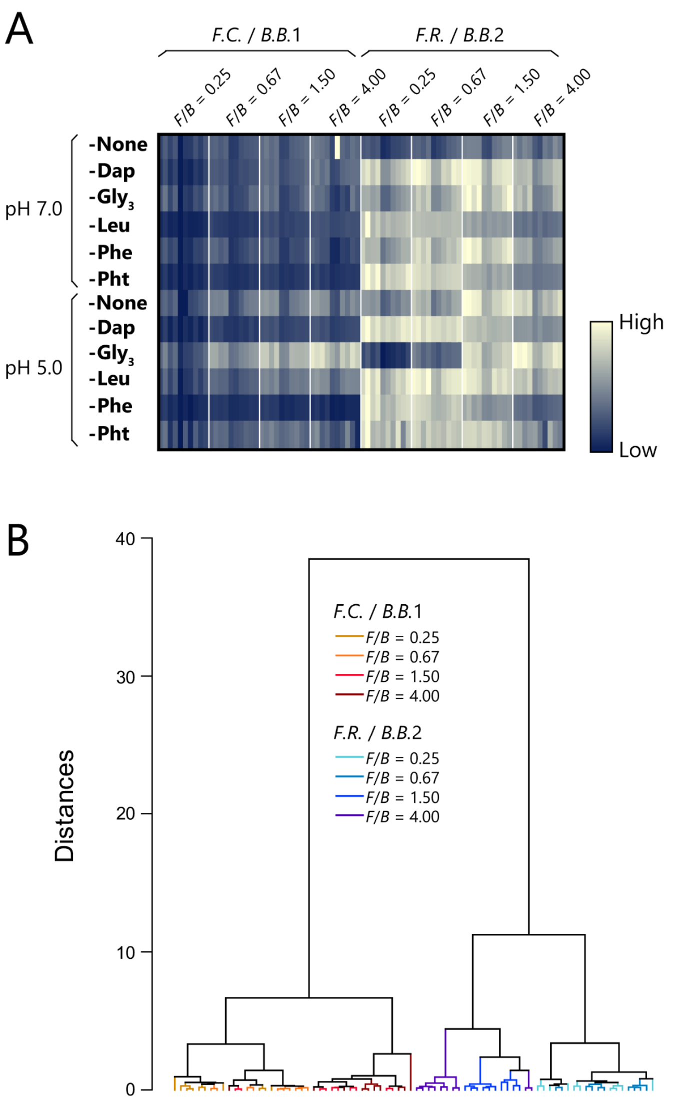

Fig. S6. Heat map (A) and the resulting HCA dendrogram (B) for the fluorescence-response patterns of the obesity model bacterial mixtures. Ten independent experimental values are shown for each analyte. In the HCA dendrogram, some analytes were not included in the cluster of the corresponding labels. Nevertheless, the LDA analysis, which allows a more accurate representation and assessment of the potential of the array for classification, yielded high accuracy as shown in Fig. 4B and Dataset 3. In other words, our array is sufficiently accurate to identify this analyte set.

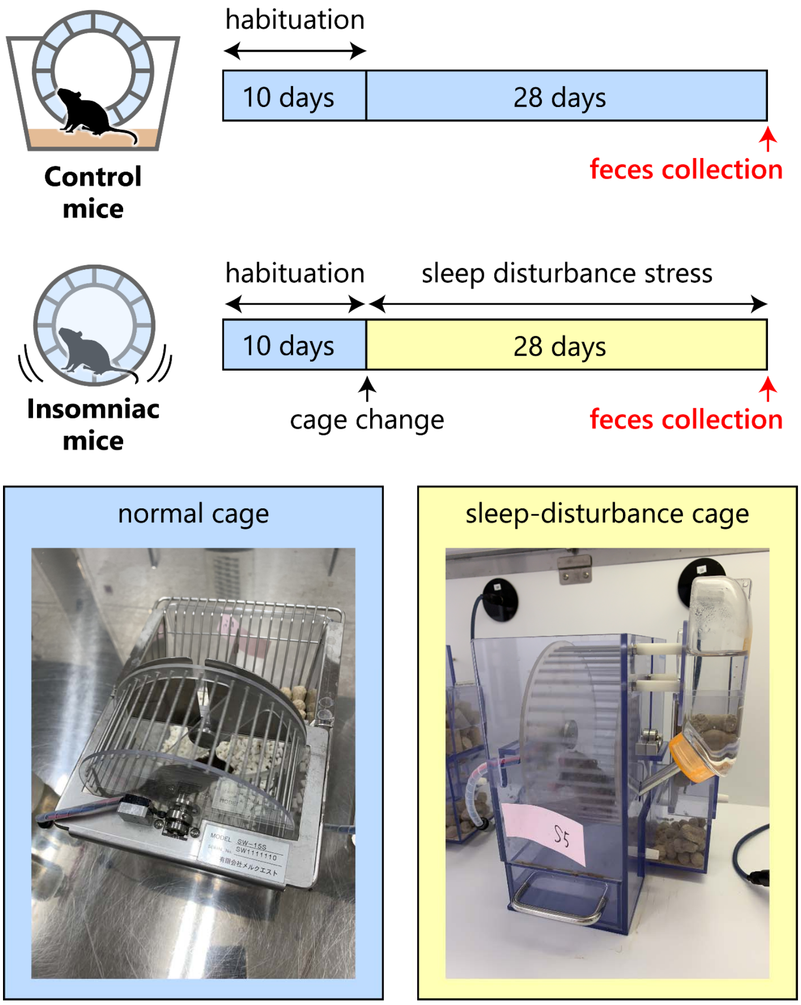

Fig. S7. Cages for the preparation of mouse feces. In a normal cage, the mouse is free to enter and exit a running wheel, while in the sleep-disturbance cage, the mouse is constantly exposed to the stress of unstable ground in an unanchored running wheel.[2,3]

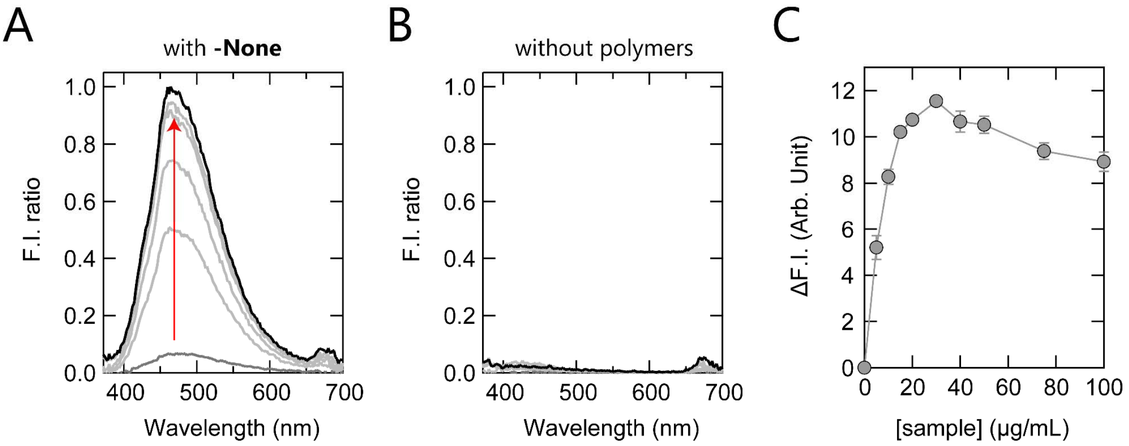

Fig. S8. Fluorescence response of **-None** to the mouse gut microbiome sample. (A, B) Fluorescence spectra (**A**: 150 nM **-None**; **B**: 0 nM **-None**) upon the addition of a mouse gut microbiome sample (healthy mouse No. 1) in 20 mM MOPS (pH = 7.0) with 150 mM NaCl; λ_{ex} = 330 nm. The background fluorescence from the sample is negligible. (C) Binding isotherms for **-None** (150 nM) upon addition of the mouse gut microbiome sample (healthy mouse No. 1) in 20 mM MOPS (pH = 7.0) with 150 mM NaCl. $\lambda_{ex}/\lambda_{em}$ = 330 nm/460 nm. Values shown represent the mean values ±1 SE from three independent experiments.

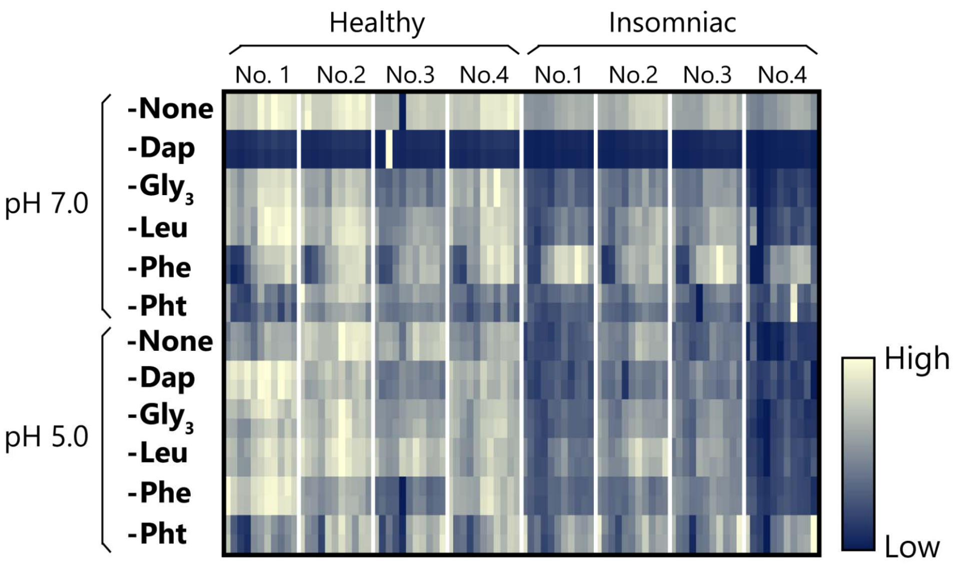

Fig. S9. Heat map of the fluorescence response patterns for the feces from the healthy and insomniac mice (20 μg/mL). For each analyte, 11 independent experimental values are shown.

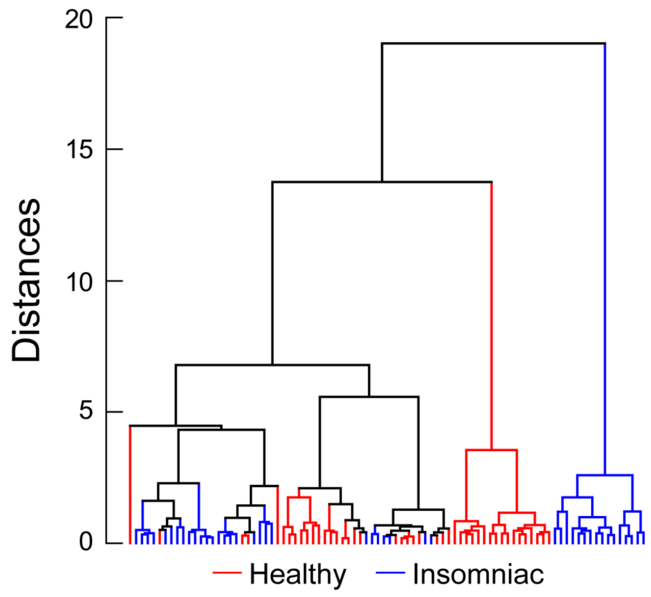

Fig. S10. HCA dendrogram of the fluorescence response patterns for the feces from healthy and insomniac mice (20 μg/mL).

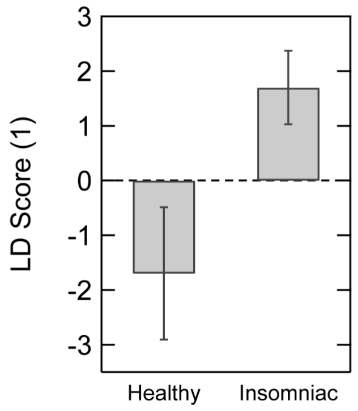

Fig. S11. Comparison of the first discriminant scores of healthy and insomniac mice (four individuals each). Mean values ± SE (n = 4; two-tailed, unpaired, Student's t-test, $p < 1.8\times10^{-3}$).

Dataset 1 (separate file). Data-set matrix of the differences in fluorescence intensity before and after the addition of gut-derived bacteria (I–I_0, OD_{600} = 0.04) generated from the chemical nose. The jackknife test afforded 99% accuracy.

Dataset 2 (separate file). Data-set matrix of the fluorescence intensity generated by the chemical nose after the addition of gut-derived bacteria (OD_{600} = 0.04). The three columns at the right indicate whether the data in the corresponding row was used as training data (denoted by "–") or test data (results of the verification are shown) in the holdout test.

Dataset 3 (separate file). Data-set matrix of the fluorescence intensity generated by the chemical nose after the addition of obesity model bacteria mixtures. The two columns at the right indicate whether the data in the corresponding row was used as training data (denoted by "–") or test data (results of the verification are shown) in the holdout test.

Dataset 4 (separate file). Data-set matrix of the fluorescence intensity generated by the chemical nose after the addition of mouse gut microbiota. The rightmost column indicates whether the data in the corresponding row was used as training data (denoted by "–") or test data (results of the verification are shown) in the holdout test.

Section 3: Synthesis

General synthetic information

Physical data were measured as follows: ^1H (400 MHz and 500 MHz) nuclear magnetic resonance (NMR) spectra were recorded using a Bruker NMR Spectrometer with DMSO-d_6, MeOD, D_2O, or $CDCl_3$ as the solvent and tetramethylsilane as the internal standard. Chemical shifts are reported in parts per million (δ), and the signals of resonances are classified as s (singlet), d (doublet), t (triplet), or m (multiplet). Analytical thin layer chromatography (TLC) was performed on Merck silica gel $60F_{254}$ precoated plates (Merck). Wakogel C-200 (particle size 75–150 μm) or Wakogel 100 C18 (Wako Pure Chemical Ind.) silica gel was used for column chromatography. Dialysis was performed using Spectra/Por 6 (cutoff 8000 kDa) tubular dialysis membranes (Spectrum Laboratories Inc.). The log P values of the head groups of the polymers were calculated using the program ALOGPs.[19]

Materials

1-(4-bromophenyl)-1,2,2-triphenylethylene, *n*-butyllithium (1.6 M in hexane), tetrahydrofuran (THF, super dehydrated), $NaSO_4$, *N*,*N*-diisopropylethylamine (DIEA), pentafluorophenyl trifluoroacetate, *N*,*N*-dimethylformamide (DMF, super dehydrated), 1H-pyrazole-1-carboxamidine hydrochloride (PCA-Cl), methanol, dimethyl sulfoxide (DMSO), piperidine, and triethylamine were purchased from Wako Pure Chemical Ind. Fmoc-glycyl-glycyl-glycine (Fmoc-$(Gly)_3$-OH), *N*-α-(9-fluorenylmethoxycarbonyl)-β-(4-pyridyl)-L-alanine (Fmoc-Ala(4-Pyri)-OH), and *N*-α,*N*-β-di(9-fluorenylmethoxycarbonyl)-L-α,β-diaminopropionic acid (Fmoc-Dap(Fmoc)-OH) were purchased from Watanabe Chemical Industries, Ltd. Polyethylene glycol-*block*-poly-L-lysine trifluoroacetate (PEG-*b*-PLL; degree of polymerization PEG: 104; degree of polymerization PLL 52; Mn = 12,000; PDI = 1.10) was purchased from Alamanda Polymers, Inc. *N*-(9-fluorenylmethoxycarbonyl)-L-proline pentafluorophenyl ester (Fmoc-Pro-OPfp), *N*-(9-fluorenylmethoxycarbonyl)-L-norleucine pentafluorophenyl ester (Fmoc-Nle-OPfp), *N*-(9-fluorenylmethoxycarbonyl)-L-phenylalanine pentafluorophenyl ester (Fmoc-Phe-OPfp), and *N*-(9-fluorenylmethoxycarbonyl)-L-leucine pentafluorophenyl ester (Fmoc-Leu-OPfp) were purchased from Santa Cruz Biotechnology. Succinic anhydride, phthalic anhydride, and 2,3-pyrazinedicarboxylic anhydride were purchased from Sigma Chemical Co. All chemicals were used without further purification.

1 TPE-OPfp

4-(1,2,2-Triphenylethenyl)benzoic acid (TPE-COOH) (**2**) was synthesized from 1-(4-bromophenyl)-1,2,2-triphenylethylene (**1**) according to a literature procedure.[20] DIEA (0.68 mL, 4.0 mmol) and pentafluorophenyl trifluoroacetate (0.51 mL, 3.0 mmol) were added to a solution of **2** (750 mg, 2.0 mmol) in DMF (30 mL) under argon, and the mixture was stirred at room temperature for 100 min. Then, ethyl acetate (150 mL) was added to the reaction mixture, before the mixture was washed four times with H_2O and then with brine. After drying the mixture over $NaSO_4$ and filtration, the organic solvent was removed from the filtrate under reduced pressure. The crude product was purified by column chromatography on silica gel using ethyl acetate–hexane (1:1, v/v) as the eluent to afford pentafluorophenyl 4-(1,2,2-triphenylethenyl)benzoate (TPE-OPfp) (**3**; 0.98 g) as a

white solid. Yield = 90%. ^{1}H NMR (500 MHz, $CDCl_3$): δ (TMS, ppm) 7.92 (d, 2 H, Ar*H*, J = 8.4 Hz), 7.20 (d, 2 H, Ar*H*, J = 8.4 Hz), 7.16–7.11 (m, 9 H, Ar*H*), 7.06–7.01 (m, 6 H, Ar*H*).

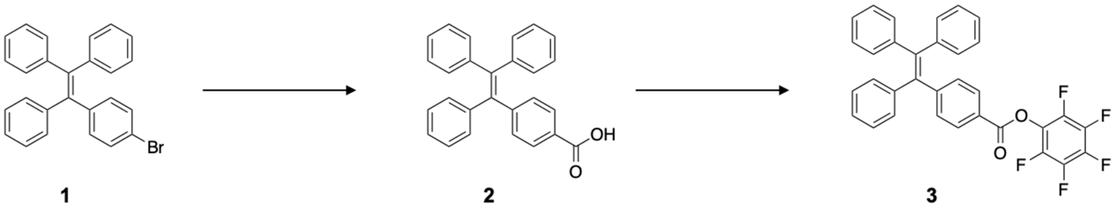

2 Fmoc-amino acid-OPfp

Fmoc-Gly$_3$-OPfp (5).

DIEA (0.75 mL, 4.4 mmol) and pentafluorophenyl trifluoroacetate (0.56 mL, 3.3 mmol) were added to a solution of Fmoc-Gly$_3$-OH (**4**) (905 mg, 2.2 mmol) in DMF (20 mL) under argon, before the mixture was stirred at room temperature for 60 min. Then, ethyl acetate (150 mL) was added to the reaction mixture, before the mixture was washed four times with H_2O and then with brine. After drying the mixture over $NaSO_4$ and filtration, the organic solvent was removed from the filtrate under reduced pressure. The crude product was suspended in a mixture of ethyl acetate (15 mL) and hexane (45 mL), and the resulting precipitate was filtered off to afford **5** (966 mg) as a white powdery solid. Yield = 76%. ^{1}H NMR (500 MHz, DMSO-d_6): δ (TMS, ppm) 8.57 (t, 1 H, N*H*, J = 5.8 Hz), 8.21 (t, 1 H, N*H*, J = 5.8 Hz), 7.89 (d, 2 H, Ar*H*, J = 7.5 Hz), 7.71 (d, 2 H, Ar*H*, J = 7.5 Hz), 7.57 (t, 1 H, N*H*, J = 6.0 Hz), 7.42 (t, 2 H, Ar*H*, J = 7.4 Hz), 7.33 (t, 2 H, Ar*H*, J = 7.4 Hz), 4.33 (d, 2 H, CH_2, J = 5.8 Hz), 4.29 (d, 2 H, CH_2, J = 7.0 Hz), 4.23 (t, 1 H, C*H*, J = 7.0 Hz), 3.80 (d, 2 H, CH_2, J = 5.8 Hz), 3.68 (d, 2 H, CH_2, J = 6.0 Hz).

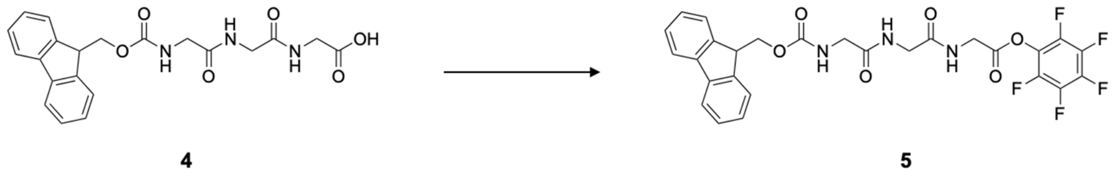

Fmoc-Ala(4-Pyri)-OPfp (7).

DIEA (1.36 mL, 8.0 mmol) and pentafluorophenyl trifluoroacetate (1.02 mL, 6.0 mmol) were added to a solution of Fmoc-Ala(4-Pyri)-OH (**6**) (1.55 g, 4.0 mmol) in DMF (40 mL) under argon, before the mixture was stirred at room temperature for 90 min. Then, ethyl acetate (200 mL) was added to the reaction mixture, before the mixture was washed four times with H_2O and then with brine. After drying the mixture over $NaSO_4$ and filtration, the organic solvent was removed from the filtrate under reduced pressure. The crude product was purified by column chromatography on silica gel with ethyl acetate–hexane (1:1, v/v) as the eluent to afford **7** (1.86 g) as a white powdery solid. Yield = 84%. ^{1}H NMR (500 MHz, DMSO-d_6): δ (TMS, ppm) 8.49 (d, 2 H, Ar*H*, J = 5.9 Hz), 8.26 (d, 1 H, N*H*, J = 7.8 Hz), 7.88 (d, 2 H, Ar*H*, J = 7.6 Hz), 7.62 (dd, 2 H, Ar*H*, J = 7.0,

5.2 Hz), 7.40 (m, 2 H, Ar*H*), 7.34 (d, 2 H, Ar*H*, *J* = 5.9 Hz), 7.29 (m, 2 H, Ar*H*), 4.82 (ddd, 1 H, C*H*, *J* = 10.5, 7.8, 5.0 Hz), 4.39 (dd, 1 H, $CH_{2}a$, *J* = 10.7, 6.9 Hz), 4.30 (dd, 1 H, $CH_{2}b$, *J* = 10.7, 6.8 Hz). 4.20 (dd, 1 H, C*H*, *J* = 6.9, 6.8 Hz), 3.27 (dd, 1 H, $CH_{2}a$, *J* = 13.8, 5.0 Hz), 3.13 (dd, 1 H, $CH_{2}b$, *J* = 13.8, 10.5 Hz).

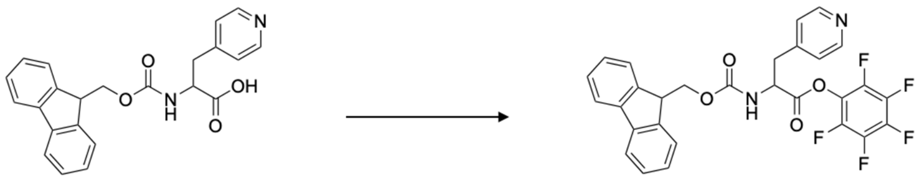

6 **7**

Fmoc-Dap(Fmoc)-OPfp (9).

DIEA (0.68 mL, 4.0 mmol) and pentafluorophenyl trifluoroacetate (0.51 mL, 3.0 mmol) were added to a solution of Fmoc-Dap(Fmoc)-OH (**8**) (1.10 g, 2.0 mmol) in DMF (20 mL) under argon, before the mixture was stirred at room temperature for 60 min. Then, ethyl acetate (150 mL) was added to the reaction mixture, before the mixture was washed four times with H_2O and then with brine. After drying the mixture over $NaSO_4$ and filtration, the organic solvent was removed from the filtrate under reduced pressure. The crude product was suspended in a mixture of ethyl acetate (10 mL) and hexane (50 mL), and the resulting precipitate was filtered off to afford **9** (1.32 g) as a white solid. Yield = 92%. ^{1}H NMR (500 MHz, DMSO-d_6): δ (TMS, ppm) 8.10 (d, 1 H, N*H*, *J* = 7.7 Hz), 7.90–7.87 (m, 4 H, Ar*H*), 7.70–7.69 (m, 2 H, Ar*H*), 7.67–7.64 (m, 2 H, Ar*H*), 7.56 (t, 1 H, N*H*, *J* = 5.9 Hz), 7.42–7.39 (m, 4 H, Ar*H*), 7.32–7.27 (m, 4 H, Ar*H*), 4.64 (m, 1 H, C*H*), 4.42–4.36 (m, 2 H), 4.34–4.30 (m, 2 H), 4.26–4.20 (m, 2 H), 3.57–3.54 (m, 2 H, CH_2).

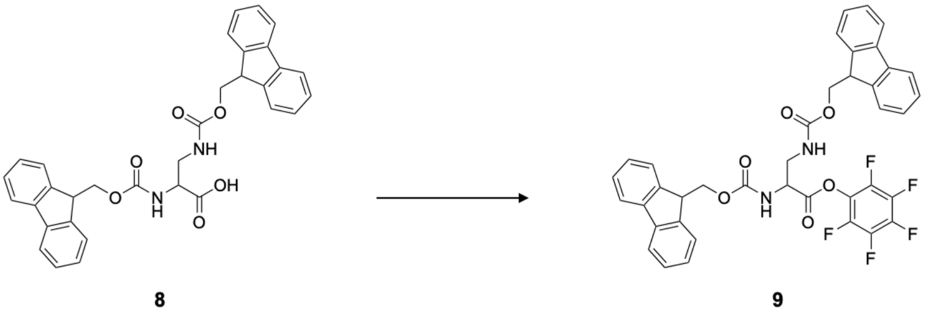

8 **9**

3 TPE-functionalized polymers

TPE-appended PEG-*b*-PLL (-None).

A solution of TPE-OPfp (**3**) (33 mg, 60 µmol) in DMF (1.5 mL) was added to a stirred solution of PEG_{104}-*b*-PLL_{52} (400 mg, 1.2 mmol with respect to the amino group) and triethylamine (500 µL, 3.6 mmol) in DMF (40 mL) under argon. The reaction mixture was stirred for 24 h at room temperature in the dark and then dialyzed twice against methanol (2 h), once against H_2O (2 h), twice against 1 mM hydrochloric acid (2 h and overnight), and once against H_2O (2 h). The final solution was lyophilized to obtain **-None**. The ^{1}H NMR spectrum (Fig. S12) confirmed that ~2.6 TPE moieties were conjugated to each PEG-*b*-PLL

based on the ratio of the integrals of the peaks of the α-C***H*** groups of the Lys backbone (δ = 3.97 ppm) to those of the 17 aromatic protons of the TPE moiety (δ = 6.9–7.1 ppm).

Guanidinylation of -None (-hA).

Guanidinylation of **-None** was carried out using a slightly modified literature procedure.[21,22] A solution of PCA-Cl (46.5 mg, 320 µmol) in methanol (1.6 mL) was added to a stirred solution of PEG_{104}-*b*-$PLL_{49.4}$/PLL-$TPE_{2.6}$ (**-None**) (30 mg, 106 µmol with respect to the amino group) and triethylamine (74 µL, 530 µmol) in methanol (4 mL) under argon. The reaction mixture was stirred for 24 h at room temperature in the dark and then dialyzed twice against methanol (2 h), once against H_2O (2 h), twice against 1 mM hydrochloric acid (2 h and overnight), and once against H_2O (2 h). The final solution was lyophilized to obtain **-hA**. The ^{1}H NMR spectrum (Fig. S13A) confirmed that almost all of the remaining Lys side chains were guanidinylated based on the disappearance of the peak of the ε-C$\boldsymbol{H}_2$ groups of the Lys side chain (δ = 2.97 ppm in MeOD).[23]

Amino acid modification of -None (-Dap, -Pro, -Nle, -Leu, -Phe, -Pyri, and -Gly_3).

The amino acid modification of **-None** was carried out using a slightly modified literature procedure.[24] A solution of Fmoc-(amino acid)-OPfp (530 µmol) in DMSO (2.7 mL) was added to a stirred solution of **-None** (30 mg, 106 µmol with respect to the amino group) and DIEA (54 µL, 320 µmol) in DMSO (3 mL) under argon. The reaction mixture was stirred for 72 h at room temperature in the dark and then dialyzed twice against methanol (2 h). After removal of the solvent under reduced pressure, DMSO (4 mL) and piperidine (1 mL) were added to the product under argon. The reaction mixture was stirred for 40 h at room temperature in the dark, and then dialyzed three times against methanol (2 h), once against H_2O (2 h), twice against 1 mM hydrochloric acid (2 h and overnight), and once against H_2O (2 h). The final solution was lyophilized to obtain the amino-acid-modified **-None**. The ^{1}H NMR spectra (Figs. S13A and S13B) confirmed that almost all of the remaining Lys side chains were modified with amino acids based on the disappearance of the peak of the ε-C$\boldsymbol{H}_2$ groups of the Lys side chain (δ = 2.97 ppm in MeOD, δ = 3.04 ppm in D_2O), as was also observed for **-hA**.

Acid anhydride modification of -None (-Suc, -Pht, and -Pyr).

Acid anhydride modification of **-None** was carried out using a slightly modified literature procedure.[25] A solution of the acid anhydride of the dicarboxylic acid (1.1 mmol) in DMSO (1.1 mL) was added to a stirred solution of **-None** (30 mg, 106 µmol with respect to the amino group) and triethylamine (150 µL, 1.1 mmol) in DMSO (4 mL) under argon. The reaction mixture was stirred for 48 h at room temperature in the dark. After adding 2.5 mL H_2O and incubating the reaction mixture for 1 h at room temperature, it was dialyzed once against 20% methanol (2 h), once against H_2O (2 h), twice against 1 mM sodium hydroxide (2 h and overnight), and once against H_2O (2 h). The final solution was lyophilized to obtain **-None** modified with acid anhydrides. The ^{1}H NMR spectra (Figs. 13A-C) confirmed that almost all of the remaining Lys side chains were modified with amino acids based on the disappearance of the peak of the ε-C$\boldsymbol{H}_2$ groups of the Lys side chain (δ = 2.97 ppm in MeOD, δ = 2.77 ppm in DMSO-d_6).[25]

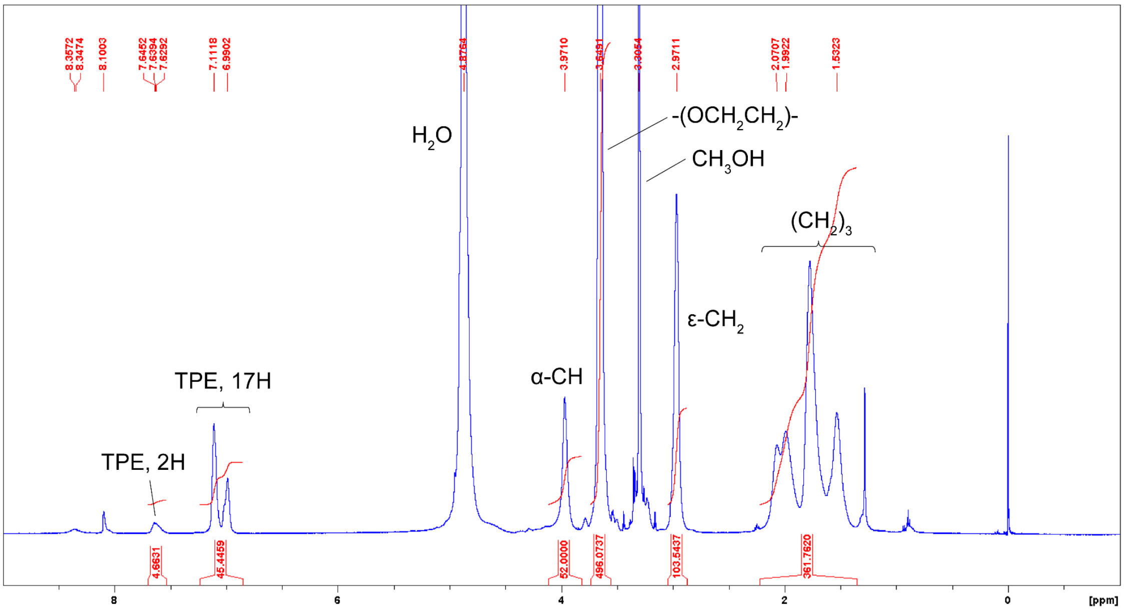

Fig. S12. 400 MHz ^{1}H-NMR spectrum of **-None** in MeOD at 25 °C.

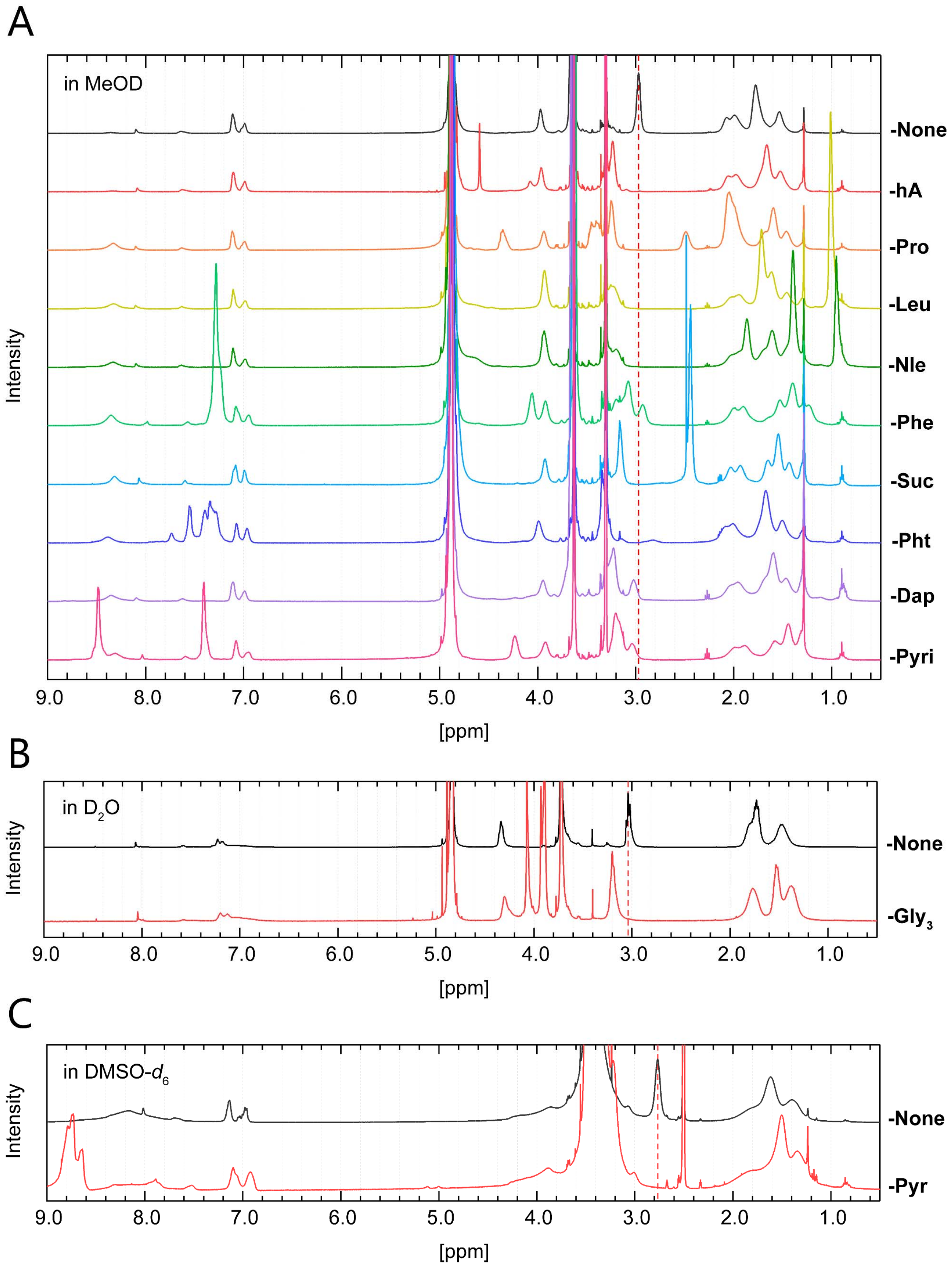

Fig. S13. ^{1}H NMR spectra of the synthesized polymers in MeOD (500 MHz: **-None**, **-hA**, **-Phe**, **-Suc**, **-Pht**; 400 MHz: **-Pro**, **-Leu**, **-Nle**, **-Dap**, **-Pyri**) (A), D_2O (400 MHz) (B), and DMSO-d_6 (400 MHz) (C) at 25 °C.

Section 4: Characterization of the polymers

As the interactions between the TPE-functionalized PEG-*b*-PLLs and bacteria may be governed primarily by electrostatic forces (Fig. 2), elucidation of the charge state of the synthesized polymers was important in order to understand and construct the chemical nose. Therefore, acid–base titration was carried out to determine the p*K*a of the polymers (Fig. S14). The titration results indicated that the functionalization of PEG-*b*-PLL with amino acids not only changed the structure of the side chains, but also greatly affected the p*K*a of the amino groups (Table S4). The introduction of amino acids with dual amino groups (**-Dap** and **-Pyri**), hydrophobic groups (**-Leu**, **-Nle**, **-Pro**), aromatic groups (**-Phe**), and tripeptides (**-Gly$_3$**) reduced the p*K*a by 2.0–3.4. These shifts in p*K*a may be at least partly attributed to the hydrophobic effects of the side chains of the introduced amino acids, as indicated in previous reports.[26,27] Similarly, acid-anhydride-modified **-Suc** and **-Pht** showed slightly higher p*K*a values than for normal carboxy groups (p*K*a = 5.8 for both). As expected, guanidinylation (**-hA**) and modification with 2,3-pyrazinedicarboxylic anhydride (**-Pyr**) significantly increased and decreased the p*K*a, respectively.

We expected that these shifts in p*K*a would affect the dispersibility of the polymers. The fluorescence intensity of each polymer was measured at various pH values (Figs. S15 and S16). The fluorescence intensities of **-None**, **-hA**, **-Dap**, and **-Pyr** were almost independent of the pH value. However, in the other polymers, large changes in fluorescence intensity were observed near their p*K*a values. The fluorescence intensity of the cationic polymers increased at pH values above their p*K*a value, while the anionic polymers showed the opposite behavior. The neutralization of the charged functional groups would reduce the electrostatic repulsion between the polymers, which may facilitate interaction between TPE moieties. As **-Dap** contains two amino groups, one of the amino groups may not be deprotonated even under weakly basic conditions (pH » 9.0), or the polymer may be able to maintain a dispersed state due to the hydrophilic amino groups even if both charges are lost under such conditions. The fluorescence of **-hA** and **-Pyr** was probably independent of the pH value because these groups were charged throughout the entire measurement range. The differences in the p*K*a values of polymers would result in a range of charge states and dispersibilities, which are closely associated with the interactions of the polymers. Therefore, it was expected that the p*K*a differences among the polymers would also contribute to the diversification of their fluorescence responses to bacteria, particularly at pH values near the p*K*a values of many of the polymers selected in this study, i.e., at pH = 5.0 and 7.0.

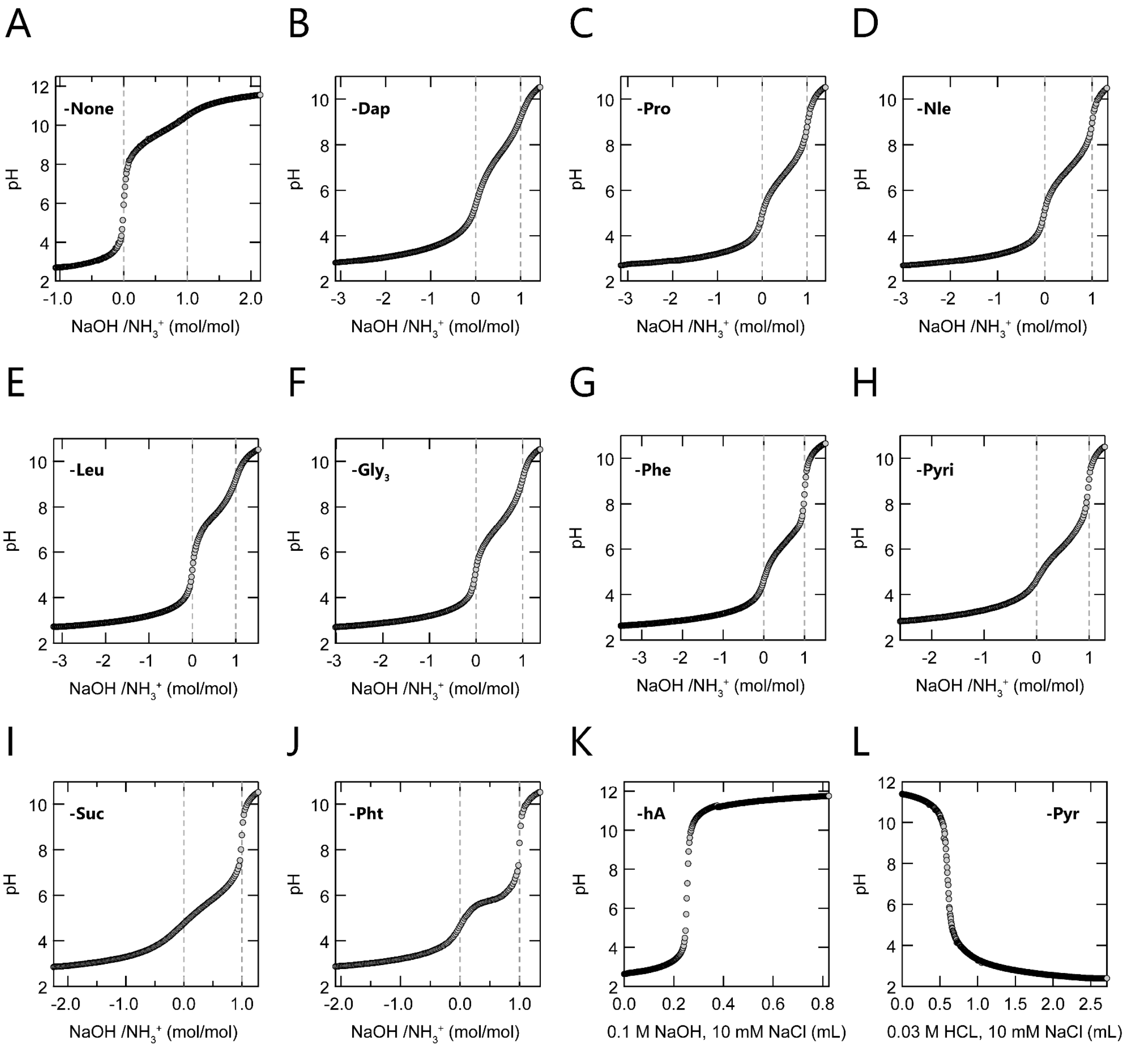

Fig. S14. Acid–base titration curves of (A) **-None**, (B) **-Dap**, (C) **-Pro**, (D) **-Nle**, (E) **-Leu**, (F) **-Gly$_3$**, (G) **-Phe**, (H) **-Pyri**, (I) **-Suc**, (J) **-Pht**, (K) **-hA**, and (L) **-Pyr**. Dotted lines indicate inflection points.

Table S4. Apparent p*K*a values of the TPE-functionalized PEG-*b*-PLLs.

Polymer	Apparent p*K*a
**-None**	9.5
**-hA**	>10
**-Dap**	7.5
**-Pro**	6.7
**-Nle**	6.9
**-Leu**	7.6
**-Gly$_3$**	7.2
**-Phe**	6.2
**-Pyri**	6.1
**-Suc**	5.8
**-Pht**	5.8
**-Pyr**	<3

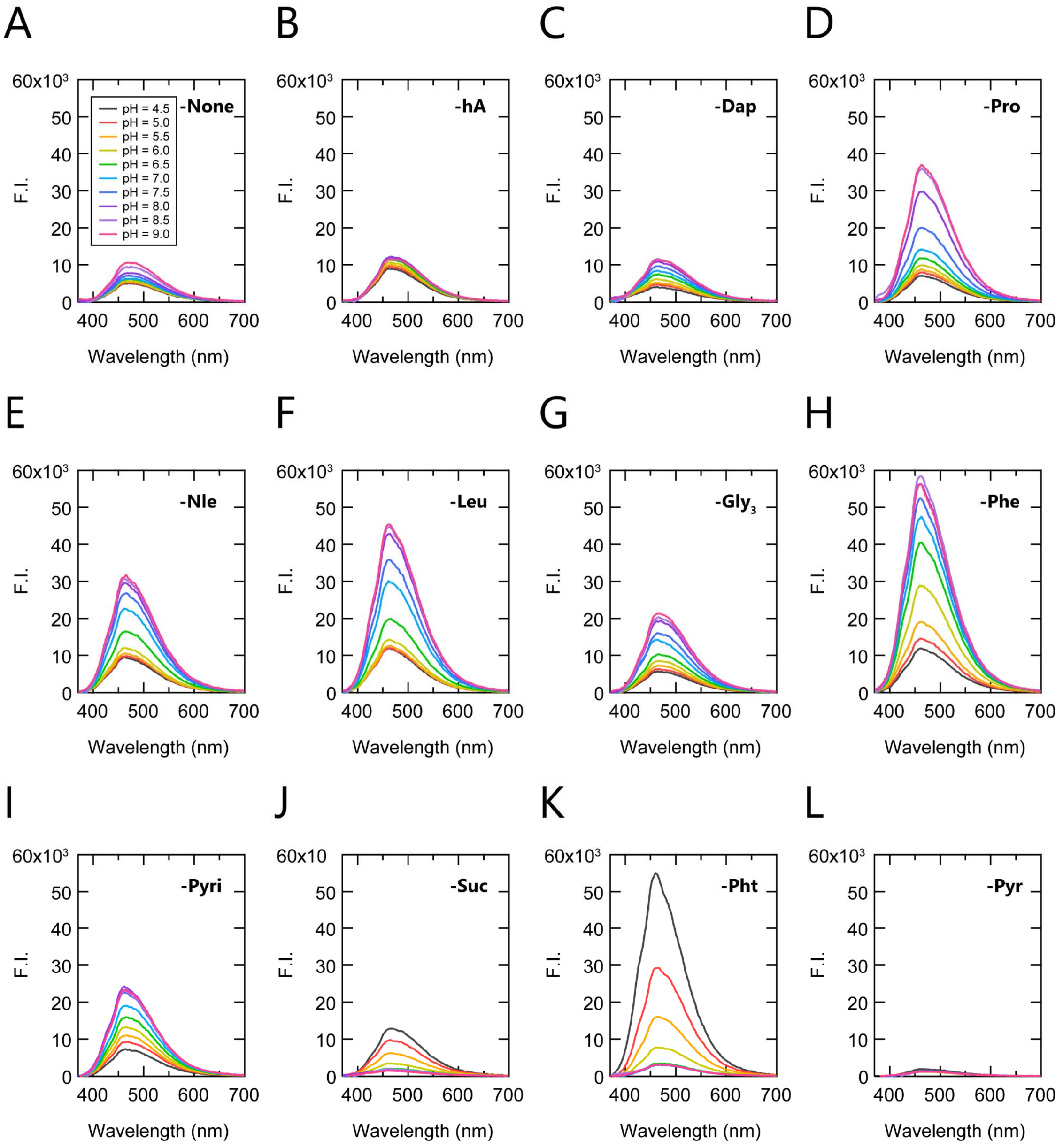

Fig. S15. pH dependence of the fluorescence spectra of (A) **-None**, (B) **-Dap**, (C) **-Pro**, (D) **-Nle**, (E) **-Leu**, (F) **-Gly$_3$**, (G) **-Phe**, (H) **-Pyri**, (I) **-Suc**, (J) **-Pht**, (K) **-hA**, and (L) **-Pyr** (150 nM) in mixed buffers of MES and MOPS (20 mM for both) (pH = 4.5 to 9.0); $\lambda_{ex}/\lambda_{em}$ = 330 nm/372-700 nm.

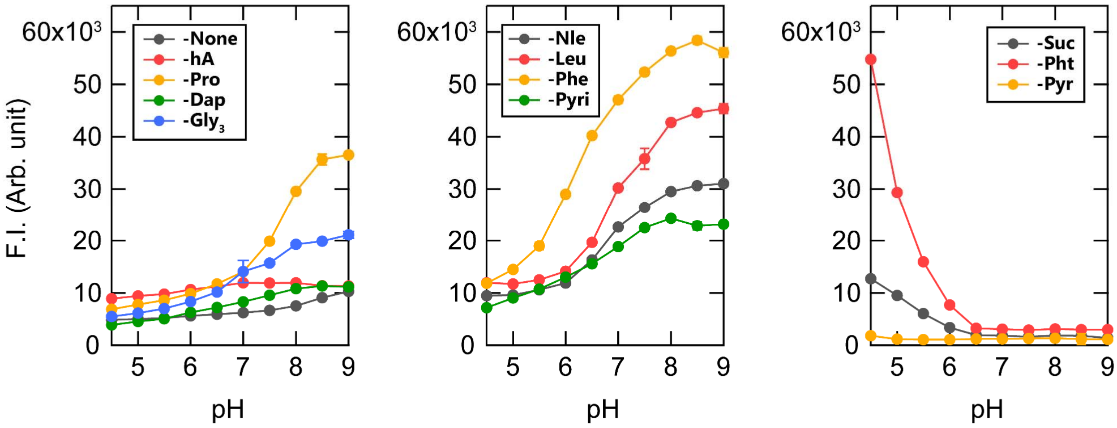

Fig. S16. The pH dependence of the fluorescence intensity of the polymers (150 nM) in mixed buffers of MES and MOPS (20 mM for both) (pH = 4.5 to 9.0); $\lambda_{ex}/\lambda_{em}$ = 330 nm/460 nm. Colored circles represent mean values ± 1 SE from three independent experiments.

Section 5: Understanding the sensing elements and reproducibility, and construction of minimal sensor systems

The array of TPE-functionalized block copolymers provided 48 elements (12 polymers × 2 pH values × 2 channels). Understanding how each element contributes to the extraction of microbial features in such a large array is important to provide guidelines for the effective selection or design of materials, as well as for discovering new applications of the arrays. Thus, we subjected all the signals from the 16 gut-derived bacteria to an unsupervised hierarchical clustering analysis (HCA), in which the calculated distance between elements corresponds to the similarities in the response patterns of these elements.[28]

In the resulting dendrogram (Fig. S17), two large clusters corresponding to the charge properties of the polymers were observed, i.e., the anionic cluster at the bottom (red background; **-Suc**, **-Pht**, and **-Pyr**) and the cationic cluster at the top (blue background; all other polymers). This result is plausible considering that microbial surfaces are commonly negatively charged, irrespective of their gram stainability,[29,30] and that electrostatic interactions should remain strong even at physiological ionic strength. Within the cationic polymer cluster, four sub-clusters were observed. One of these subclusters corresponded to hydrophobic and cationic polymers (**-Pyri**, **-Leu**, **-Nle**, and **-Phe**) at pH = 7.0; hydrophobic interactions with, e.g., lipopolysaccharides may therefore play a role in their unique affinity. The remaining clusters consisted of hydrophilic polymers with (**-Pro**, **-Dap** and **-Gly$_3$**) or without (**-None** and **-hA**) amino acid modifications at pH = 7.0, and all the cationic polymers at pH = 5.0, suggesting that differences in the solution pH are also a key factor. It should be noted that, although macromolecules that bear multiple guanidium ions can interact strongly with biomolecules through multivalent salt-bridge formation,[31] unexpectedly, guanidination of TPE-functionalized PEG-*b*-PLL (**-hA**) did not contribute strongly to the recognition of bacterial differences.

These tendencies were also similar in loading plots (Fig. S18). For example, in the plots of PC1 vs. PC2, we observed (i) clusters of anionic polymers, (ii) clusters of cationic polymers with hydrophobic amino acids at pH = 7.0, and (iii) clusters of cationic polymers without amino acids at pH = 7.0. These plots also suggest that the data of two channels (Ch1 and Ch2) co-varied. Therefore, their contribution should be lower than those of polymer structures and pH values, although the cost of obtaining data in different channels is quite low. In fact, even with Ch1 alone, the validation tests showed high reliability (99% in a leave-one-out cross-validation test and 100% in a holdout test). In addition, the loading plots showed that the difference in pH values particularly affected PC2 and PC4, e.g., the points corresponding to pH = 7.0 appeared to be distributed in the positive direction in PC2 and in the negative direction in PC4. These results indicate that the differences in both polymer structure and pH value are important for providing cross-reactivity to gut-derived bacteria.

In summary, (i) the charge state and (ii) hydrophobicity of the polymers, as well as (iii) the solution pH, contributed to the diversification of the fluorescence responses to bacteria, demonstrating the effectiveness of our polymer design and choice of solution conditions for sensing gut-derived bacteria. Based on the HCA results, the subsequent studies used a lower number of combinations while maintaining a sufficiently high performance of the sensor elements in models of gut microbiota associated with obesity (Fig. 4), real mouse microbiota (Fig. 5), and the others (*vide infra*), i.e., six polymers (**-None**, **-Dap**, **-Gly$_3$**, **-Leu**, **-Phe**, and **-Pht**), the two pH values, and Ch1. The six polymers were selected evenly from the five clusters observed in Fig. S17, with the expectation of efficiently generating fluorescence responses with low similarity, i.e., diverse responses. We decided to continue to use two different pH values (pH = 5.0 and 7.0) as pH differences played an important role in diversifying

responses (Fig. S17). In TPE-functionalized polymers, we found that differences in detection channels contributed little to obtaining different information, and hence, Ch1 was chosen as it produces higher fluorescence intensity.

In order to gain a deeper insight into the effects of the pH value, we attempted to discriminate gut-derived bacteria with the addition of weakly basic conditions (pH = 9.0) using a chemical nose consisting of the above six polymers (**-None**, **-Dap**, **-Gly$_3$**, **-Leu**, **-Phe**, and **-Pht**) and detection using Ch1 (Fig. S19; for the raw data, see Dataset 5). As inferred from the HCA analysis (Fig. S17), the distribution of clusters in the LDA plots obtained at pH = 5.0 and pH = 7.0 was different, and most of the clusters were well separated (the jackknife test afforded 100% accuracy in both cases). In contrast, at pH = 9.0, some of the clusters overlap, and the accuracy based on the jackknife test was 92%. This decrease in accuracy was probably due to an increase in fluorescence intensity prior to bacterial addition, which should be associated with deprotonation of the cationic polymers (Figs. S15 and S16). These results suggest that the selection of a weakly acidic (pH = 5.0) and a neutral (pH = 7.0) conditions is sufficiently effective in the present TPE-functionalized block-copolymers for gut-derived bacteria sensing.

In addition, we compared the results of the same experiment performed on different days (E1 and E2) in order to examine the reproducibility of our chemical noses that consist of six polymers (**-None**, **-Dap**, **-Gly$_3$**, **-Leu**, **-Phe**, and **-Pht**), two pH values (pH = 5.0 and 7.0), and Ch1 (Fig. S20; for the raw data, see Dataset 6). Fluorescence pattern data of 16 kinds of labels (8 gut-derived bacteria × 2 experiments) analyzed by LDA showed that the clusters of the same bacteria were very close to each other even if the experimental dates were different. Importantly, when a holdout test was conducted using the dataset obtained by E1 as training data and the dataset obtained by E2 as test data, 100% accuracy was obtained (48 out of 48). Therefore, our chemical noses offer a robust evaluation system that is capable of reproducibly distinguishing gut-derived bacteria.

While the construction of chemical noses with high discrimination potentials is important, for practical applications, it is also significant to find minimum components that exhibit sufficient discriminatory power. Therefore, the accuracy of chemical noses composed of two polymers in identifying the 16 intestinal bacterial strains was comprehensively tested (Table S5). Some pairs that roughly satisfy the factors mentioned in the previous paragraph, such as **-Dap**/**-Phe** and **-Dap**/**-Pyri**, provided 100% accuracy in the jackknife test for the identification of intestinal bacterial strains. In the score (1) and score (2) plot obtained using the **-Dap**/**-Phe** combination, the clusters of *P.E.*1 and *B.B.*3 and those of *F.E.* and *F.L.*1 overlapped partially, but they were separated in the score (3) and score (5) space (Fig. S21). In addition, this combination also provided 97% accuracy in the holdout test. These results suggest that dual amino groups (**-Dap**) and an aromatic ring (**-Phe**) are particularly suitable structural features for the recognition of the bacterial surfaces. Thus, we have demonstrated that a wide range of chemical noses can be constructed, ranging from minimal systems with sufficient reliability (Fig. S21) to large systems with further potential for identifying more bacteria (Fig. 3).

The combination of solely **-None** and **-Dap**, which afforded 100% accuracy for gut-derived bacteria in the jackknife test above, also achieved high accuracy for the mouse fecal samples (93% for the jackknife test and 97% for the holdout test). Cluster analysis of the response patterns generated by the chemical nose composed of six polymers (Fig. 5) showed a low correlation between the responses of these two polymers (Fig. S22). These results suggested that this minimal sensor could possibly be useful for a wide range of applications, from identifying bacterial strains to distinguishing mouse gut microbiota.

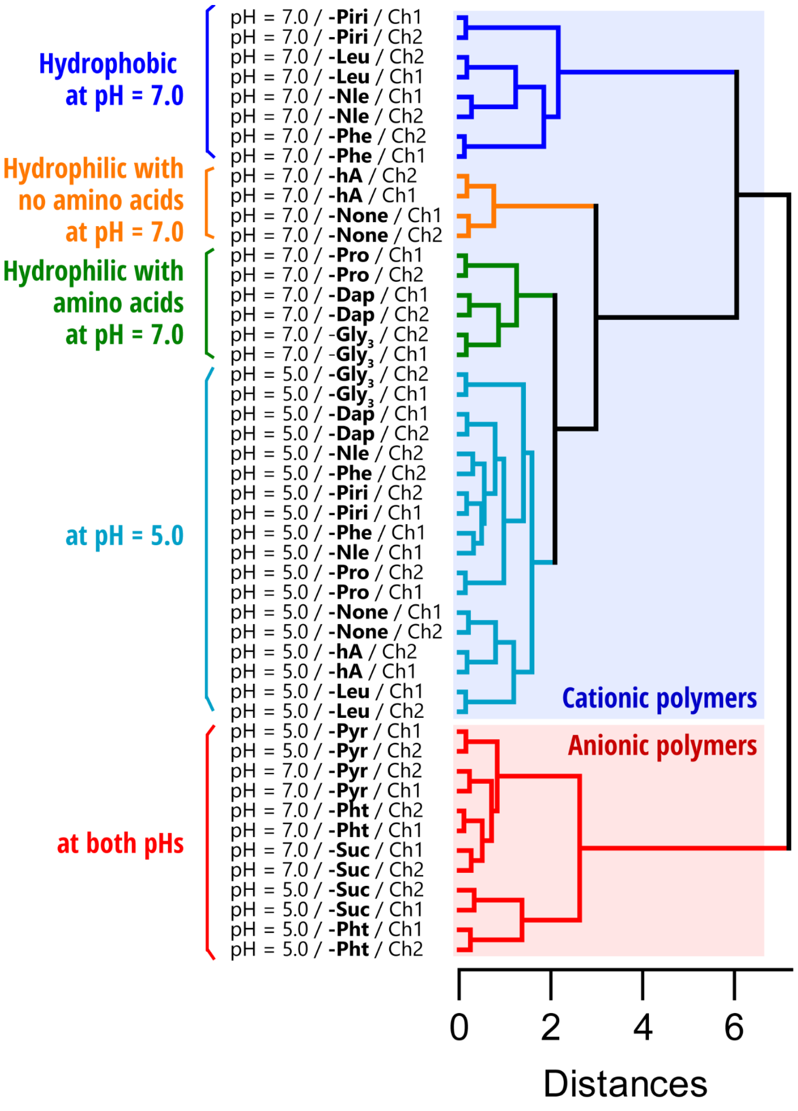

Fig. S17. Clustering analysis of the sensing elements. A hierarchical clustering dendrogram was created based on the Euclidean distances using the Ward method and a standardized dataset (48 elements × 16 gut-derived bacteria × 11 replicates).

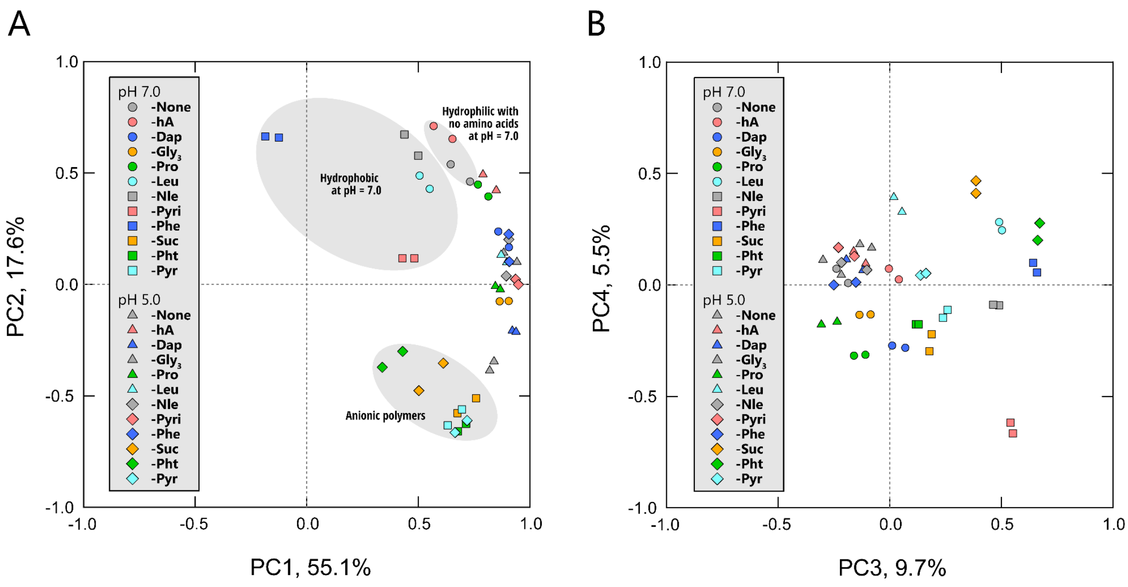

Fig. S18. Loading plots for the intestinal bacterial strains (OD_{600} = 0.04) using the array consisting of 12 TPE-functionalized PEG-*b*-PLLs. (A) Score (1) vs. score (2); (B) score (3) vs. score (4); for each pH/polymer, two points corresponding to Ch1 and Ch2 were plotted.

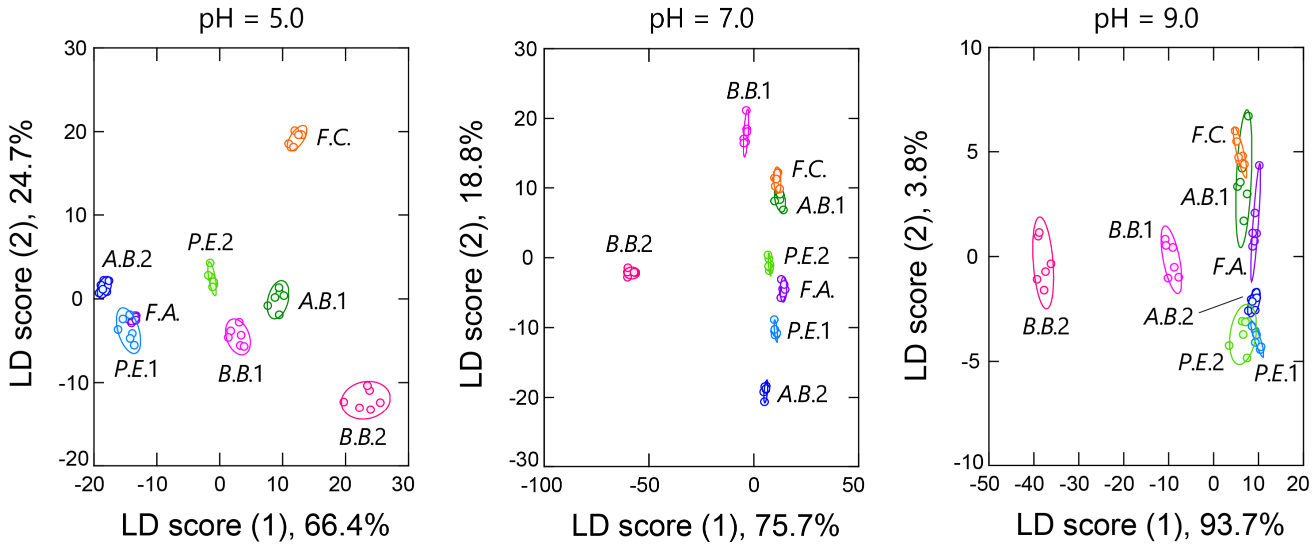

Fig. S19. The effects of the pH value on two-dimensional LDA score plots for gut-derived bacteria (OD_{600} = 0.04) obtained from the array consisting of six polymers (**-None**, **-Dap**, **-Gly$_3$**, **-Leu**, **-Phe**, and **-Pht**) and detection using Ch1. The ellipsoids represent the confidence intervals (±1 SD) for each analyte. For each analyte, six independent experimental values are shown.

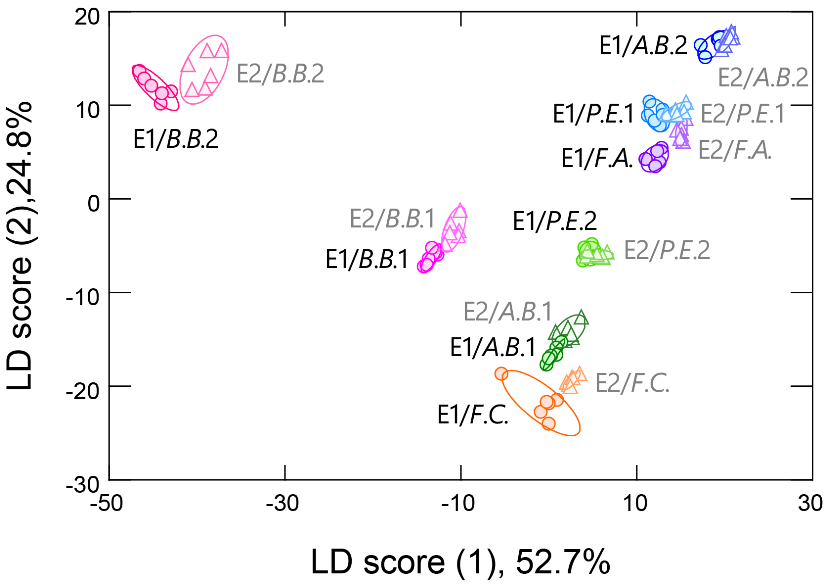

Fig. S20. The effects of carrying out the same experiment on different dates on the two-dimensional LDA score plot for gut-derived bacteria (OD_{600} = 0.04) obtained from the array consisting of six polymers (**-None**, **-Dap**, **-Gly$_3$**, **-Leu**, **-Phe**, and **-Pht**), two pH values (pH = 5.0 and 7.0), and detection using Ch1. The ellipsoids represent the confidence intervals (±1 SD) for each analyte. For each analyte, six independent experimental values are shown.

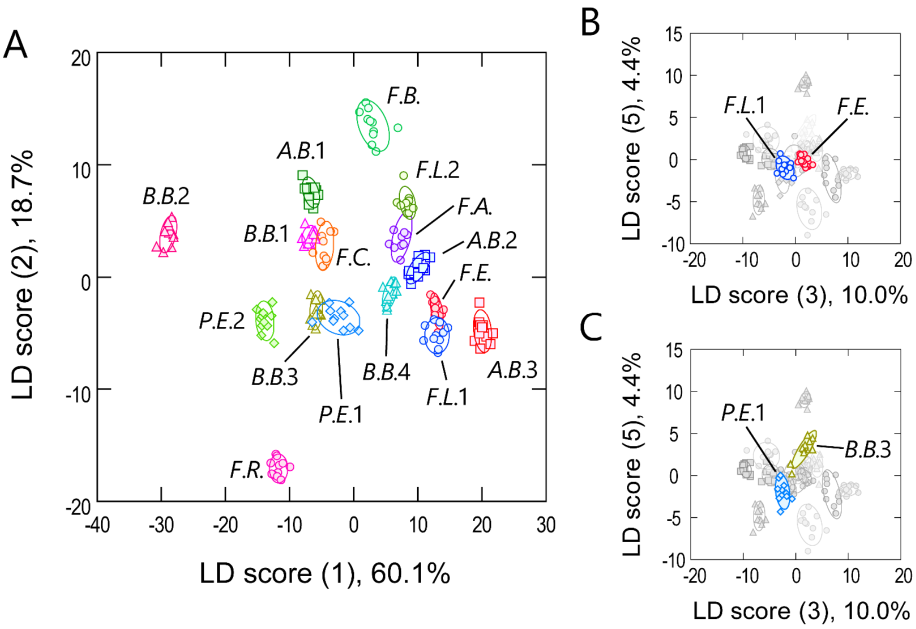

Fig. S21. Two-dimensional LDA score plots for gut-derived bacteria (OD_{600} = 0.04) obtained from the array consisting of **-Dap** and **-Phe**. The ellipsoids represent the confidence intervals (±1 SD) for each analyte. (A) Score (1) vs. score (2). (B,C) Score (3) vs. score (5); *F.E.* and *F.L.*1 (B) or *B.B.*3 and *P.E.*1 (C) are highlighted.

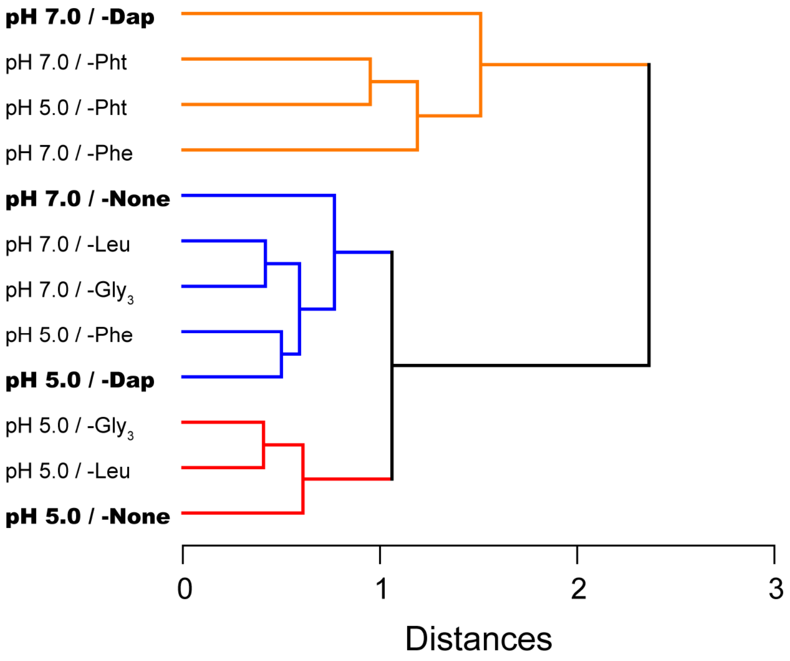

Fig. S22. Clustering analysis of the sensing elements. A hierarchical clustering dendrogram was created based on the Euclidean distances using the Ward method and a standardized dataset (12 elements × 8 mouse gut microbiota × 11 replicates).

Table S5. Classification accuracy of the chemical noses consisting of two polymers for the identification of gut-derived bacteria.

Selected polymers												%correct	
**-None**	**-hA**	**-Dap**	**-Pro**	**-Nle**	**-Leu**	**-Gly$_3$**	**-Phe**	**-Pyri**	**-Suc**	**-Pht**	**-Pyr**		
■	■											98	
■		■										100	**-None + -Dap**
■			■									97	
■				■								98	
■					■							97	
■						■						98	
■							■					97	
■								■				99	
■									■			97	
■										■		97	
■											■	97	
	■	■										98	
	■		■									98	
	■			■								97	
	■				■							98	
	■					■						98	
	■						■					98	
	■							■				99	
	■								■			98	
	■									■		97	
	■										■	97	
		■	■									98	
		■		■								99	
		■			■							99	
		■				■						98	
		■					■					100	**-Dap + -Phe**
		■						■				100	**-Dap + -Pyri**
		■							■			99	
		■								■		98	
		■									■	99	
			■	■								97	
			■		■							95	
			■			■						99	
			■				■					97	
			■					■				98	
			■						■			94	
			■							■		94	
			■								■	95	
				■	■							99	
				■		■						98	
				■			■					99	
				■				■				99	
				■					■			99	
				■						■		98	
				■							■	97	
					■	■						98	
					■		■					98	
					■			■				97	
					■				■			94	
					■					■		94	
					■						■	97	
						■	■					98	

						99
						99
						97
						97
						99
						96
						97
						97
						99
						99
						99
						82
						92
						90

Dataset 5 (separate file). Data-set matrix of the fluorescence intensity generated by the chemical nose after the addition of gut-derived bacteria (OD_{600} = 0.04) at three different pH values.

Dataset 6 (separate file). Data-set matrix of the fluorescence intensity of the same experiment carried out on different dates generated by the chemical nose after the addition of gut-derived bacteria (OD_{600} = 0.04). The rightmost column indicates whether the data in the corresponding row was used as training data (denoted by "–") or test data (results of the verification are shown) in the holdout test.

Section 6: Identification of *Escherichia coli* strains

Fig. 3B shows that an array of TPE-functionalized block-copolymers was able to distinguish different strains of *Escherichia coli* (*P.E.*1 and *P.E.*2). Bacteria strains belonging to the same species are populations of progeny that have differentiated from a single bacterium and have essentially the same genetic characteristics as the parent bacterium. Therefore, distinguishing between strains using conventional metagenomic methods such as sequencing of 16S ribosomal RNA gene amplicons is generally difficult. To further investigate the applicability of our chemical nose, we attempted to identify eight different strains of *Escherichia coli*, including *P.E.*1 (= DH5α) and *P.E.*2 (= JM109) (Table S2), as well as those shown in Figs 3 and 4 using a chemical nose composed of six polymers (**-None**, **-Dap**, **-Gly$_3$**, **-Leu**, **-Phe**, and **-Pht**), (for the raw data, see Dataset 7).

Statistical analysis of the fluorescence response patterns (Fig. S23A) using HCA showed that each strain was clustered to some extent (Fig. S23B). LDA showed that the clusters of all of the strains, except those of Rosetta2 (DE3) and Rosetta-gami B (DE3), were distributed without overlap in the two-dimensional space (Fig. S24A). The clusters of these two strains were almost separated in the plot of score (4) vs. score (5) (Fig. S24B). The jackknife test and the holdout test provided high accuracy (100% and 91%, respectively). Although several reports of the identification of *Escherichia coli* strains have been published,[32–34] this study represents one of the largest numbers of strains to be identified so far. At this stage, it is not clear what differences among the *Escherichia coli* strains were recognized by our chemical nose, and more systematic studies are needed to clarify the discrimination mechanism. Nevertheless, our system may open new avenues for the easy identification of industrially useful bacterial and pathogenic variants.

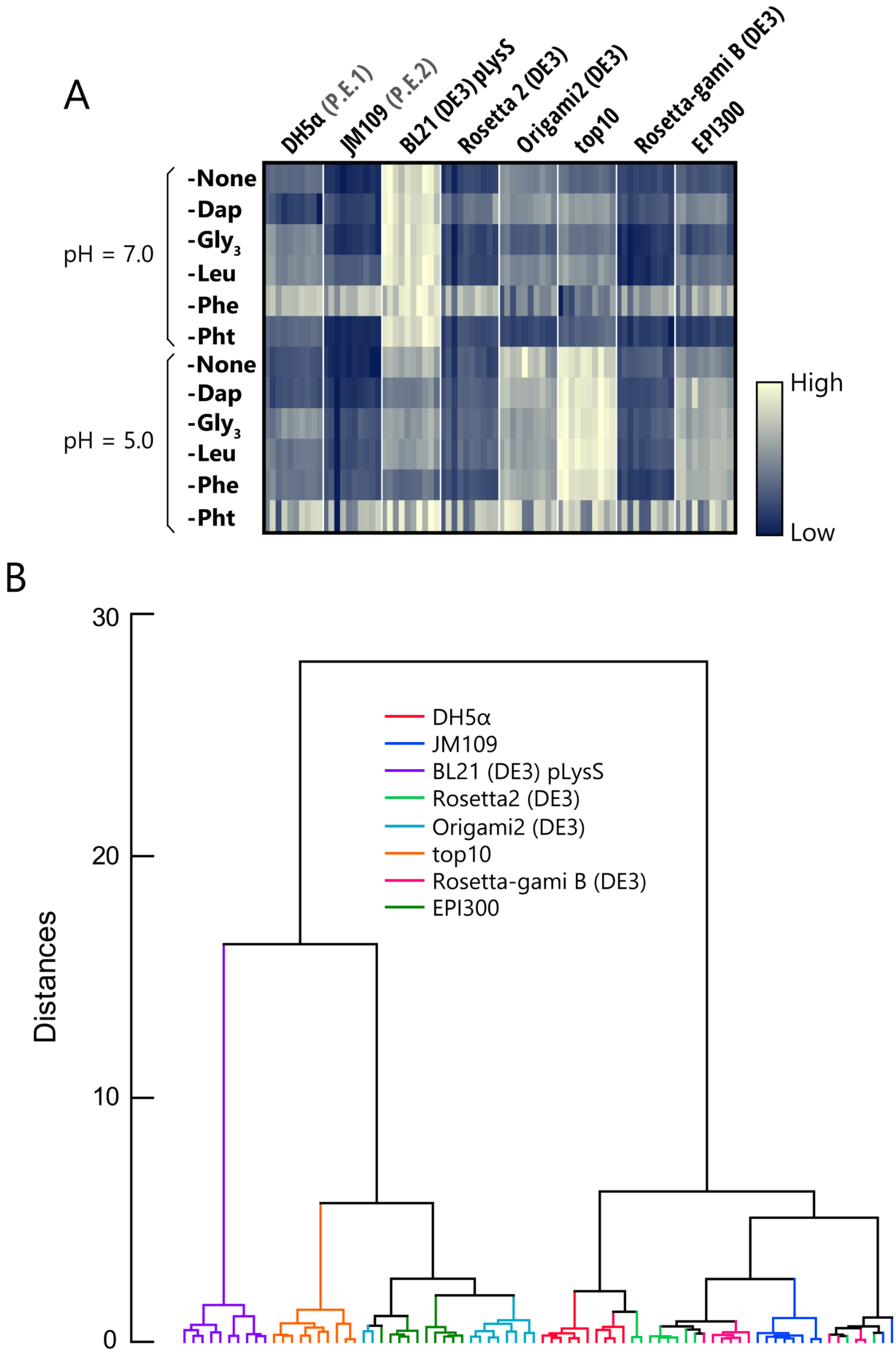

Fig. S23. Heat map (A) and the resulting HCA dendrogram (B) of the fluorescence response patterns of *Escherichia coli* strains (OD600 = 0.04). For each analyte, 10 independent experimental values are shown.

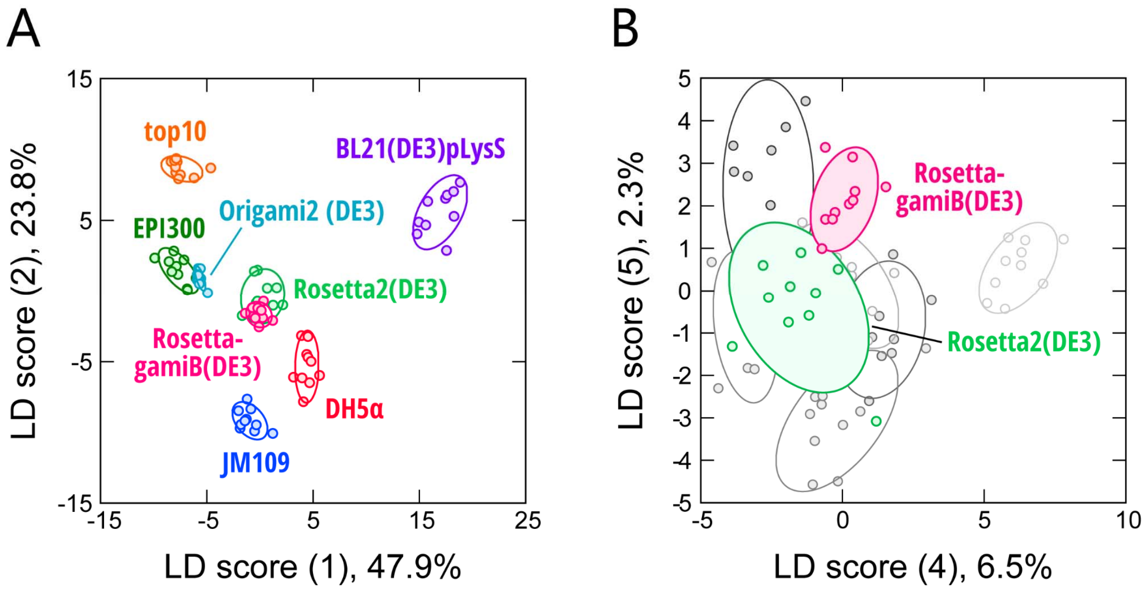

Fig. S24. Two-dimensional LDA score plots for *Escherichia coli* strains (OD_{600} = 0.04). For each analyte, ten independent experimental values are shown. The ellipsoids represent the confidence intervals (±1 SD) for each analyte. (A) Score (1) vs. score (2). (B) Score (4) vs. score (5); Rosetta2 (DE3) and Rosetta-gami B (DE3) are highlighted.

Dataset 7 (separate file). Data-set matrix of the fluorescence intensity generated by the chemical nose after the addition of *Escherichia coli* strains. The rightmost column indicates whether the data in the corresponding row was used as training data (denoted by "–") or test data (results of the verification are shown) in the holdout test.

Section 7: Identification of model microbiome samples spiked with trace amounts of gut-derived bacteria

In Fig. 4, the mixtures of two gut-derived bacteria were analyzed as model microbiome samples. Such compositional differentiation should be valuable in considering the application of chemical noses to target changes in the overall gut-microbiome compositions associated with diseases such as the *F/B* ratio. From another perspective, changes in the abundance of certain bacteria in the gut microbiome may also be significant. For example, *Clostridium spp. (*phylum *Firmicutes)* and *Blautia spp. (*phylum *Firmicutes)* can vary from a few percent to about 15% of the total gut bacteria at the population level.[35] Members of the genus *Bacteroides* (phylum *Bacteroidetes*) can account for as much as 30% of the human gut microbiome.[36] The ability to detect changes in such particular classes of bacteria may lead to the creation of unique applications. Therefore, we have attempted to take a step forward on this issue, although that this step remains preliminary at this point.

First, we examined the sensitivity of our chemical noses before testing them under conditions close to those of the actual scenario. The two-dimensional LDA score plot of two gut-derived bacteria (*F.C.* and *B.B.*4) with different OD_{600} values obtained by a chemical nose composed of six polymers (**-None**, **-Dap**, **-Gly$_3$**, **-Leu**, **-Phe**, and **-Pht**), two pH values (pH = 5.0 and 7.0), and Ch1 showed cluster overlap at low OD_{600} values (e.g., OD_{600} = 0.0003 and 0.0010 *B.B.*4) (Fig. S25A; for the raw data, see Dataset 8). Consistently, the confusion matrix of the jackknife test showed that misclassification occurred in *B.B.*4 with OD_{600} = 0.0003 and 0.0010 (Fig. S25B). Therefore, the detection limit of this method is estimated to be in the range of OD_{600} = 0.0010-0.0030.

In the present study, model samples were prepared wherein two gut-derived bacteria (*F.C.* and *B.B.*4) were spiked with 7.9%, 14,6%, and 20.5% of the fecal microbiome, based on the estimated detection limit and peak responses of these gut-derived bacteria shown in Fig. 2B (OD_{600} = 0.035) and the peak response of the real microbiome shown in Fig. S8 (30 µg/mL); in other words, *F.C.* or *B.B.*4 with OD_{600} = 0.001, 0002, and 0.003 were spiked against the mouse microbiome at 10 µg/mL. As noted above, these bacteria concentration may be roughly within the range of realistic microbiome variations. Statistical analysis of the fluorescence response patterns (Fig. S26A and Dataset 9) obtained by the chemical nose using LDA revealed that the seven clusters were clearly separated with marginal overlap of '0.002 *F.C.*' and '0.003 *F.C.*' in the two-dimensional space (Fig. S26B). Consistent with this result, the jackknife test afforded a relatively high discriminant accuracy of 93%. In addition, a difference in the change in the cluster position was observed between *F.C.* and *B.B.*4 with increasing bacterial concentration. The clusters of *F.C.* moved in the positive direction of score (1) and score (2), and those of *B.B.*4 moved in the positive direction of score (1). This result suggests that improved chemical-nose systems may be able to detect changes in abundance of specific bacterial taxa in the microbiome.

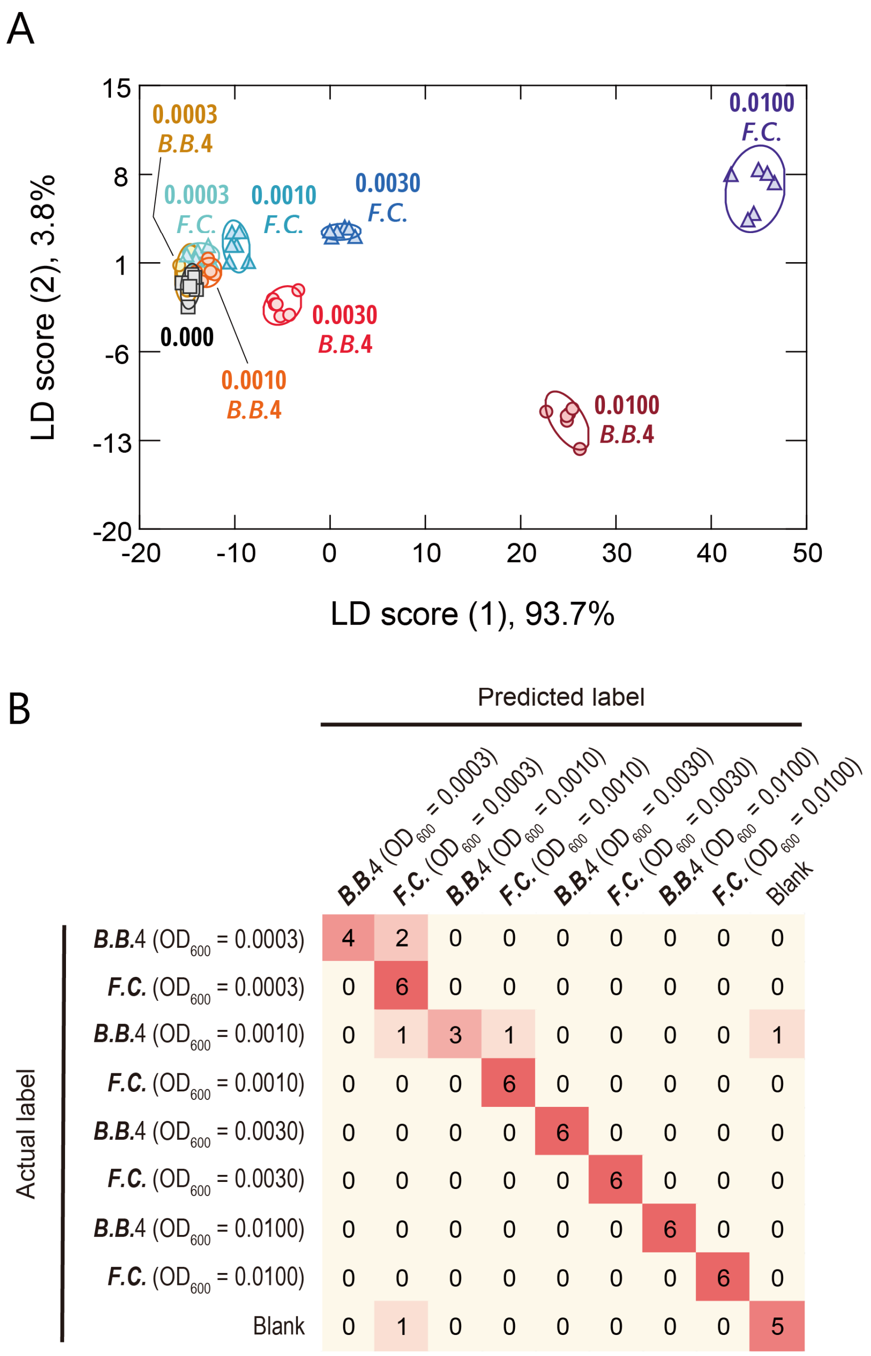

Fig. S25. Optical-pattern recognition of gut-derived bacteria with different OD_{600} values. (A) Two-dimensional discriminant score plot, wherein the ellipsoids represent the confidence intervals (±1 SD) for each analyte. For each analyte, six independent experimental values are shown. (B) Confusion matrix of the jackknife test.

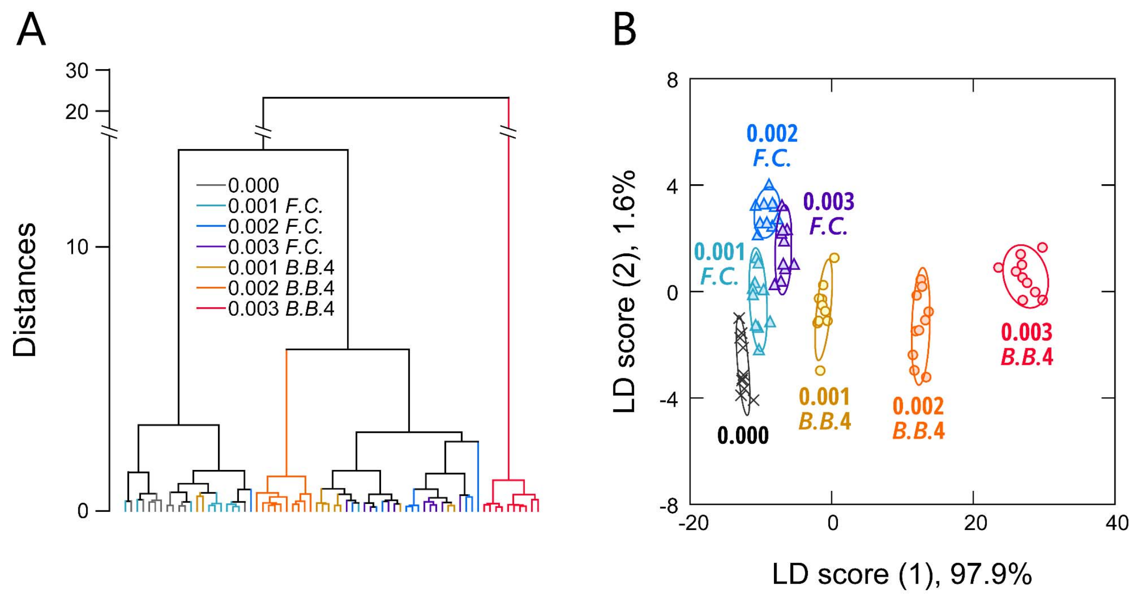

Fig. S26. Optical-pattern recognition of model microbiome samples spiked with trace amounts of gut-derived bacteria. (A) HCA dendrogram of the patterns of fluorescence intensity after the addition of real mouse microbiome samples spiked with trace amounts of gut-derived bacteria. Ten independent experimental values are shown for each analyte. For example, '0.001 *F.C.*' is a sample of 10 µg/mL mouse microbiome spiked with *F.C.* at OD_{600} = 0.001. (B) A resulting two-dimensional discriminant score plot, wherein the ellipsoids represent the confidence intervals (±1 SD) for each analyte

Dataset 8 (separate file). Data-set matrix of the fluorescence intensity generated by the chemical nose after the addition of gut-derived bacteria with different OD_{600} values.

Dataset 9 (separate file). Data-set matrix of the fluorescence intensity after the addition of model microbiome samples spiked with trace amounts of gut-derived bacteria generated from the chemical nose.

Section 8: References

1 H. Kusada, K. Kameyama, X.-Y. Meng, Y. Kamagata and H. Tamaki, *Sci. Rep.*, 2017, **7**, 18087.

2 K. Miyazaki, N. Itoh, S. Ohyama, K. Kadota and K. Oishi, *PLoS One*, 2013, **8**, e55452.

3 E. N. Minakawa, K. Miyazaki, K. Maruo, H. Yagihara, H. Fujita, K. Wada and Y. Nagai, *Neurosci. Lett.*, 2017, **653**, 362–369.

4 K. Oishi, M. Konishi, Y. Murata and N. Itoh, *Biochem. Biophys. Res. Commun.*, 2011, **412**, 396–400.

5 M. Matsumoto, R. Kibe, T. Ooga, Y. Aiba, S. Kurihara, E. Sawaki, Y. Koga and Y. Benno, *Sci. Rep.*, 2012, **2**, 233.

6 B. Zacchetti, P. Smits and D. Claessen, *Front. Microbiol.*, 2018, **9**, 943.

7 A. Schwiertz, G. L. Hold, S. H. Duncan, B. Gruhl, M. D. Collins, P. A. Lawson, H. J. Flint and M. Blaut, *Syst. Appl. Microbiol.*, 2002, **25**, 46–51.

8 C. Liu, S. M. Finegold, Y. Song and P. A. Lawson, *Int. J. Syst. Evol. Microbiol.*, 2008, **58**, 1896–1902.

9 Y. A. Warren, K. L. Tyrrell, D. M. Citron and E. J. C. Goldstein, *J. Clin. Microbiol.*, 2006, **44**, 2416–2422.

10 M. M. Taylor, *J. Gen. Microbiol.*, 1972, **71**, 457–463.

11 M.-C. Domingo, A. Huletsky, M. Boissinot, K. A. Bernard, F. J. Picard and M. G. Bergeron, *Int. J. Syst. Evol. Microbiol.*, 2008, **58**, 1393–1397.

12 K. H. Schleifer, J. Kraus, C. Dvorak, R. Kilpper-Bälz, M. D. Collins and W. Fischer, *Syst. Appl. Microbiol.*, 1985, **6**, 183–195.

13 S. M. Naser, K. E. Hagen, M. Vancanneyt, I. Cleenwerck, J. Swings and T. A. Tompkins, *Int. J. Syst. Evol. Microbiol.*, 2006, **56**, 355–360.

14 M. A. Bakir, M. Sakamoto, M. Kitahara, M. Matsumoto and Y. Benno, *Int. J. Syst. Evol. Microbiol.*, 2006, **56**, 1639–1643.

15 H. Hayashi, K. Shibata, M. A. Bakir, M. Sakamoto, S. Tomita and Y. Benno, *Int. J. Syst. Evol. Microbiol.*, 2007, **57**, 1323–1326.

16 Y. Watanabe, F. Nagai, M. Morotomi, H. Sakon and R. Tanaka, *Int. J. Syst. Evol. Microbiol.*, 2010, **60**, 1864–1869.

17 S. Sakata, M. Kitahara, M. Sakamoto, H. Hayashi, M. Fukuyama and Y. Benno, *Int. J. Syst. Evol. Microbiol.*, 2002, **52**, 1945–1951.

18 S. Sakata, C. S. Ryu, M. Kitahara, M. Sakamoto, H. Hayashi, M. Fukuyama and Y. Benno, *Microbiol. Immunol.*, 2006, **50**, 1–10.

19 I. V. Tetko, J. Gasteiger, R. Todeschini, A. Mauri, D. Livingstone, P. Ertl, V. A. Palyulin, E. V. Radchenko, N. S. Zefirov, A. S. Makarenko, V. Y. Tanchuk and V. V. Prokopenko, *J. Comput. Aided Mol. Des.*, 2005, **19**, 453–463.

20 Z. Song, Y. Hong, R. T. K. Kwok, J. W. Y. Lam, B. Liu and B. Z. Tang, *J. Mater. Chem. B Mater. Biol. Med.*, 2014, **2**, 1717–1723.

21 M. S. Bernatowicz, Y. Wu and G. R. Matsueda, *J. Org. Chem.*, 1992, **57**, 2497–2502.

22 I. Tsogas, T. Theodossiou, Z. Sideratou, C. M. Paleos, H. Collet, J. C. Rossi, B. Romestand and A. Commeyras, *Biomacromolecules*, 2007, **8**, 3263–3270.

23 S. Kudo and Y. Nagasaki, *Macromol. Rapid Commun.*, 2015, **36**, 1916–1922.

24 S. Tomita, S. Ishihara and R. Kurita, *ACS Appl. Mater. Interfaces*, 2019, **11**, 6751–6758.

25 N. Shimada, M. Nakayama, A. Kano and A. Maruyama, *Biomacromolecules*, 2013, **14**, 1452–1457.
26 D. W. Urry, S. Peng and T. Parker, *J. Am. Chem. Soc.*, 1993, **115**, 7509–7510.
27 D. W. Urry, S. Peng, D. C. Gowda, T. M. Parker and R. D. Harris, *Chem. Phys. Lett.*, 1994, **225**, 97–103.
28 Z. Li, J. R. Askim and K. S. Suslick, *Chem. Rev.*, 2019, **119**, 231–292.
29 T. J. Silhavy, D. Kahne and S. Walker, *Cold Spring Harb. Perspect. Biol.*, 2010, **2**, a000414.
30 N. Malanovic and K. Lohner, *Biochim. Biophys. Acta*, 2016, **1858**, 936–946.
31 R. Mogaki, P. K. Hashim, K. Okuro and T. Aida, *Chem. Soc. Rev.*, 2017, **46**, 6480–6491.
32 R. L. Phillips, O. R. Miranda, C.-C. You, V. M. Rotello and U. H. F. Bunz, *Angew. Chem. Int. Ed.*, 2008, **47**, 2590–2594.
33 J. Han, H. Cheng, B. Wang, M. S. Braun, X. Fan, M. Bender, W. Huang, C. Domhan, W. Mier, T. Lindner, K. Seehafer, M. Wink and U. H. F. Bunz, *Angew. Chem. Int. Ed.*, 2017, **56**, 15246–15251.
34 J.-Y. Yang, X.-D. Jia, X.-Y. Wang, M.-X. Liu, M.-L. Chen, T. Yang and J.-H. Wang, *Chem. Commun.* , 2020, **56**, 1717–1720.
35 S. Nishijima, W. Suda, K. Oshima, S.-W. Kim, Y. Hirose, H. Morita and M. Hattori, *DNA Res.*, 2016, **23**, 125–133.
36 R. Nagpal, S. Wang, L. C. Solberg Woods, O. Seshie, S. T. Chung, C. A. Shively, T. C. Register, S. Craft, D. A. McClain and H. Yadav, *Front. Microbiol.*, 2018, **9**, 2897.
